# Does Chronic Kidney Disease Predict Stroke Risk Independent of Blood Pressure? A Systematic Review and Meta-Regression

**DOI:** 10.1161/STROKEAHA.119.025442

**Published:** 2019-10-09

**Authors:** Dearbhla M. Kelly, Peter M. Rothwell

**Affiliations:** From the Center for Prevention of Stroke and Dementia, Nuffield Department of Clinical Neurosciences, John Radcliffe Hospital, University of Oxford, United Kingdom.

**Keywords:** blood pressure, cardiovascular disease, epidemiological studies, glomerular filtration rate, hypertension, stroke

## Abstract

Chronic kidney disease is strongly associated with stroke with various purported mechanisms proposed. Low glomerular filtration rate appears to be a risk factor for stroke independent of cardiovascular risk factors in epidemiological studies, but there has been no systematic assessment of the impact of more complete adjustment for blood pressure on the association.

**Methods—:**

We did a systematic review to February 2018 (MEDLINE/EMBASE) for cohort studies or randomized controlled trials that reported stroke incidence in adults according to baseline estimated glomerular filtration rate. Study and participant characteristics and relative risks (RR) were extracted. Estimates were combined using a random-effects model. Heterogeneity was assessed by x^2^ statistics and *I*^2^ and by subgroup strata and meta-regression.

**Results—:**

We identified 168 studies reporting data on 5 611 939 participants with 115 770 stroke outcomes. Eighty-five studies (3 417 098 participants; 72 996 strokes) provided adequate data for meta-analysis of estimated glomerular filtration rate and stroke risk. Incident stroke risk was increased among participants with estimated glomerular filtration rate <60 mL/min per 1.73 m^2^ (RR=1.73; 95% CI, 1.57–1.90; *P*<0.001), but there was substantial heterogeneity between studies (*P*<0.0001; *I*^2^, 78.5%). Moreover, the association was reduced after adjustment for cardiovascular risk factors, with progressive attenuation on more thorough adjustment for hypertension: single baseline blood pressure measure (RR=1.63; CI, 1.34–1.99; *P*<0.001); history or treated hypertension (RR=1.35; CI, 1.24–1.46; *P*<0.001); multiple blood pressure measurements over months to years (RR=1.10; CI, 1.02–1.18; *P*=0.01).

**Conclusions—:**

The association between chronic kidney disease and stroke appears to be highly dependent on the method of adjustment for hypertension. The apparently independent relationship between chronic kidney disease and stroke may be confounded by their shared association with long-term prior blood pressure.

## Abstract 2

Supplemental Digital Content is available in the text.

Chronic kidney disease (CKD) is a global health burden with an estimated prevalence of 11% to 13%.^1^ In patients with CKD, compared with the general population, cardiovascular disease such as stroke is more frequent and severe.^2^ CKD prevalence varies from 20% to 35% in patients with acute ischemic stroke^3,4^ and from 20% to 46% in patients with acute intracerebral hemorrhage.^5,6^ Even in patients with mild to moderate CKD, the incidence of cardiovascular death is much higher than the incidence of kidney failure.^7^

The mechanisms of increased stroke risk in CKD remain unclear with possible contributions from traditional risk factors such as diabetes mellitus and atrial fibrillation, and nontraditional risk factors such as uremia, chronic inflammation, abnormal calcium metabolism.^8^ There is conflicting epidemiological evidence about whether low estimated glomerular filtration rate (eGFR) is a risk factor for stroke independent of traditional cardiovascular risk factors. In a pooled analysis of 22 634 participants from community-based longitudinal studies including the Atherosclerosis Risk in Communities study, Cardiovascular Health Study, Framingham Heart Study, and Framingham Offspring Study, an excess risk of stroke with a lower eGFR was not statistically significant after adjusting for traditional cardiovascular risk factors.^9^ However, in larger meta-analyses, in which multivariate-adjusted relative risks were pooled, the risk of incident stroke increased by 43% in patients with an eGFR below 60 mL/min per 1.73 m^2^ with risk of stroke increasing 7% for every 10 mL/min per 1.73 m^2^ decrease in GFR.^10,11^ These associations were not different across subtypes of stroke, sex and varying prevalence of cardiovascular risk factors.

Increased vascular risk in individuals with CKD may, however, be mostly attributable to coexistent or prior hypertension, the most prevalent comorbidity in individuals with CKD, occurring in 67% to 92% of patients.^12^ It is the leading modifiable risk factor for stroke in the general population, regardless of age, sex, or stroke subtype.^13^ However, the extent of its contribution to increased stroke risk in patients with CKD in the context of other potential mechanisms is not clear.

We aimed to determine if there is still an independent relationship between CKD and stroke risk after adjusting for traditional risk factors, particularly with more complete adjustment for confounding by blood pressure. We hypothesize that the association between CKD and stroke is not independent of long-term blood pressure burden.

## Methods

The authors declare that all supporting data are available within the article (and in the online-only Data Supplement).

### Data Sources and Searches

Using the same protocol, we updated an earlier systematic review and meta-analysis of randomized controlled trials and cohort studies that had estimated the association between GFR and the risk of stroke.^11^ Our study protocol was registered prospectively on PROSPERO (CRD42019126862) and conformed with PRISMA guidelines (Preferred Reporting Items for Systematic Reviews and Meta-Analyses). We searched MEDLINE (2013–February 2018) and EMBASE (2013–February 2018) databases using a search strategy developed by a specialized librarian that combined text word and medical subject headings without language restrictions (Materials and Appendix Table I in the online-only Data Supplement).

### Study Selection

We included all RCTs and cohort studies that measured GFR at baseline and reported quantitative estimates with a measure of precision (or original data which allowed their calculation) of the risk of incident or recurrent stroke in relation to GFR. GFR had to be either estimated using a validated formula (Cockcroft-Gault, modification of diet in renal disease [MDRD], CKD epidemiology collaboration [CKD-EPI]), measured directly, approximated from urinary creatinine clearance or estimable from serum creatinine. The outcome of interest was symptomatic stroke confirmed by physician examination, hospital record review, or identified from data-linkage of administrative records. Eligible articles were evaluated for overlap based on geographic setting, study period, sample size, and outcome. We excluded cross-sectional and case–control studies due to the greater risk of bias than in prospective cohort studies, studies where GFR was measured using nonvalidated methods, studies that had mostly participants with end-stage renal disease (by history of dialysis or an eGFR <15 mL/min per 1.73 m^2^), studies where outcomes were measured by self-reports or proxy reports and studies that reported radiological but clinically silent stroke disease. Studies that used slightly varying eGFR intervals were included if they were otherwise comparable.

### Data Abstraction and Quality Assessment

Key descriptive and quantitative data were recorded for study characteristics, participants, exposures, and outcomes. We collected details of the year of study publication, location, size, and duration. Abstracted participant characteristics included age, sex, race, and the prevalence of diabetes mellitus, known vascular diseases, smoking, and hypertension. We also noted if participants were recruited at a time of high stroke risk, including around an acute coronary event, coronary revascularization procedure, or carotid arterial intervention. We recorded the GFR, the method of measurement, and the units of quantification used. We then extracted data for the relative risk (RR), odds, rate or hazard ratio of stroke associated with each specified GFR and noted whether reported strokes were fatal or nonfatal, incident or recurrent, as well as the subtype of stroke (hemorrhagic, ischemic, or unspecified). We obtained effect estimates from both the unadjusted (or minimally adjusted) and the most fully adjusted model reported, noting which variables the model had adjusted for. The SE of the estimate was also extracted or estimated from the reported 95% CI. We assessed the quality of cohort studies using the Newcastle–Ottawa Scale.^14^

### Statistical Analysis

The leading outcome of interest was the risk of stroke in patients with an eGFR <60 mL/min per 1.73 m^2^. When articles provided estimates based on both the MDRD and CKD-EPI equations, we used estimates from the CKD-EPI equation as these result in more accurate risk prediction for adverse outcomes compared with the MDRD study equation.^15^ We converted RRs associated with averaged GFR to their natural logarithms and combined log RRs and standard errors using the DerSimonian and Laird method in a random-effects model. A fixed-effect model was also used for comparison with the random effects model on the overall risk estimate. Reported *P* values were 2-sided, with significance set at <0.05. Heterogeneity among included studies was assessed by x^2^ statistics and the *I*^2^ test. We regarded heterogeneity as possibly unimportant when the *I*^2^ value was <40% and considerable when >75%.^16^ We used subgroup analyses and meta-regression to explore sources of inconsistency and heterogeneity. Subgroups were prespecified and included study characteristics (study design, size, location, and duration of follow-up), participant characteristics (age, sex, race, prevalence of diabetes mellitus, hypertension, smoking, atrial fibrillation, undergoing cardiac, or carotid intervention, GFR defined by CKD stage) and characteristics of stroke recorded (subtype, severity, and whether incident or recurrent).

To evaluate the impact of the type of hypertension adjustment on effect estimates, we derived and reported results according to a hierarchy of adjustment from worst to best: no adjustment; adjustment for single (or few) blood pressure readings at one sitting at study entry alone; composite adjustment for a single blood pressure measurement or historical or treated hypertension; adjustment for historical or treated hypertension; and adjustment for multiple blood pressure readings generally over months or years.

Publication bias was assessed by visual examination of funnel plots. For all analyses, we used Stata software version 13 (Stat Corp, College Station, TX).

## Results

**Figure 1.**
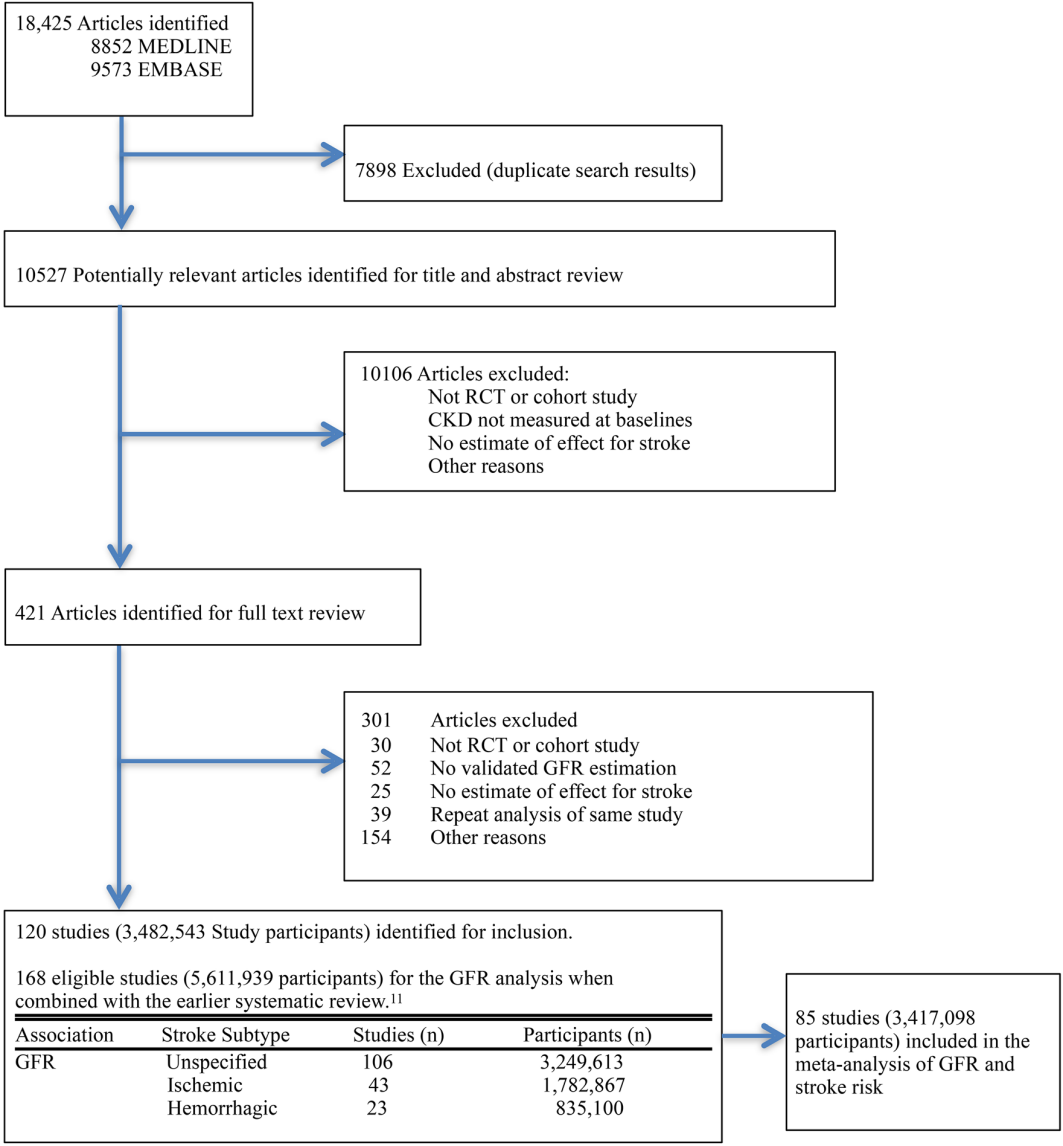
Identification and inclusion of study reports of chronic kidney disease (CKD) and stroke risk. GFR indicates glomerular filtration rate; and RCT, randomized controlled trial.

In addition to the earlier review of 61 studies,^11^ we identified a further 107 eligible studies that assessed stroke risk in CKD, resulting in 168 eligible studies reporting 115 770 strokes in 5 611 939 participants. Eighty-five studies (3 417 098 participants) provided appropriate quantitative data to be included in the meta-analysis of GFR and stroke risk (Figure 1 and Materials and Appendix Table II in the online-only Data Supplement). The data were derived from 67 cohort studies and 18 randomized controlled trials. Characteristics of the included studies and randomized trials are described in Materials and Appendix Tables II and III in the online-only Data Supplement. GFR was most commonly estimated using the MDRD formula (42 studies; 49.4%) followed by the CKI-EPI formula (18 studies; 21.2%) and then the Cockcroft-Gault equation (17 studies; 20%). Fifty-three of the included studies (62.4%) were published from 2010 onwards. The majority of studies were based in Asia (31 studies; 36.5%) followed by North America (24 studies; 28.2%).

In total, there were 72 996 stroke outcome events including 36 085 which were not classified by pathological subtype (unspecified), 35 143 ischemic, and 1758 hemorrhagic strokes. Sixty-three studies (74.1%) reported unspecified stroke types, 29 studies (34.1%) reported ischemic strokes, and 17 studies (20%) reported hemorrhagic strokes (Materials and Appendix Table III in the online-only Data Supplement). Pooling unadjusted results from the random-effects model showed that incident stroke risk was increased among patients with an eGFR <60 mL/min per 1.72 m^2^ (RR=1.72; 95% CI, 1.57–1.90; *P*<0.001; Materials and Appendix Figure I in the online-only Data Supplement). In pooled multivariate adjusted analysis, usually after adjustment for age, sex and cardiovascular risk factors, this risk association attenuated to an RR of 1.36 (1.29–1.43; *P*<0.001; Materials and Appendix Figure II in the online-only Data Supplement).

**Figure 2.**
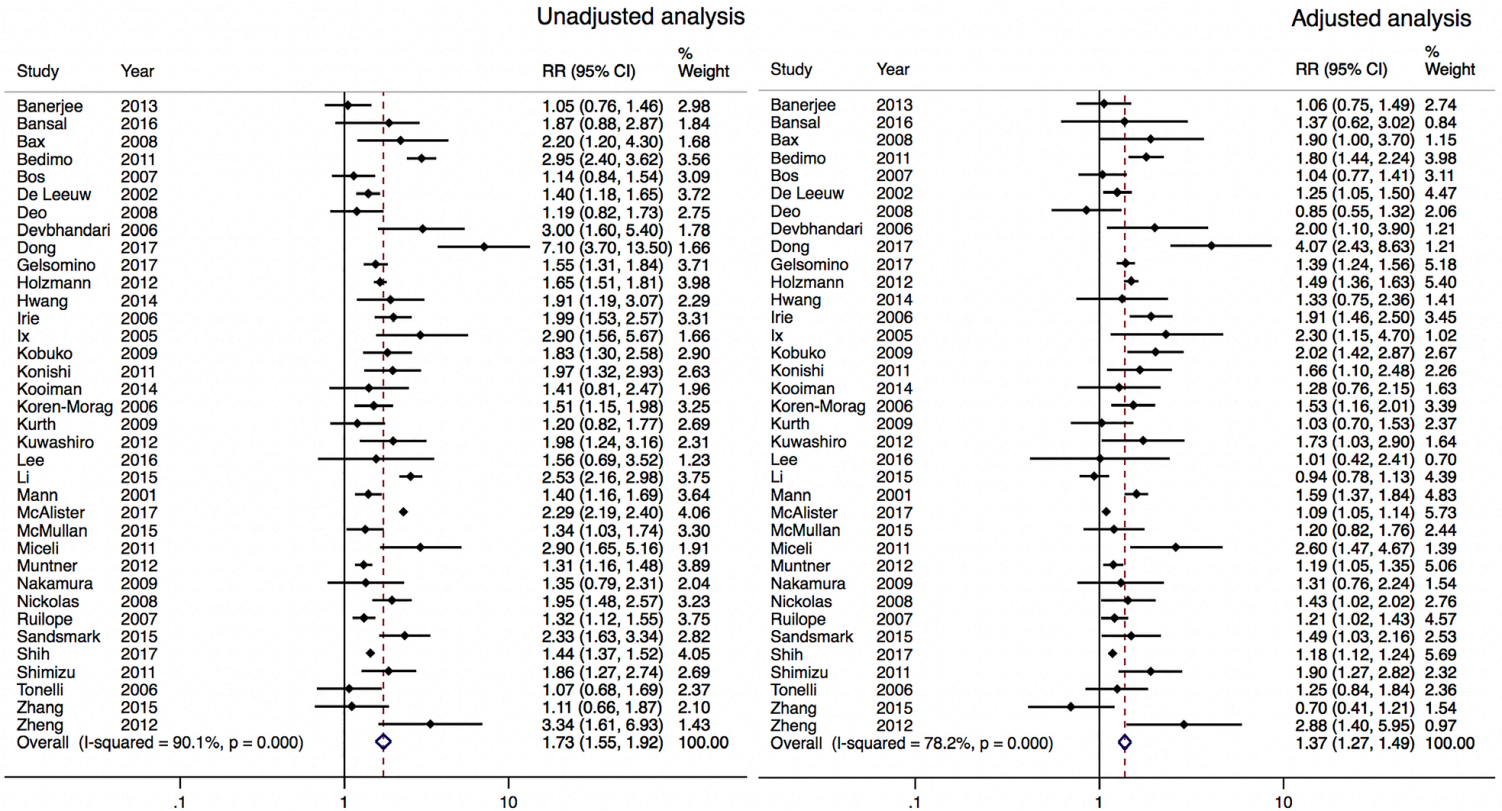
Unadjusted and adjusted risk ratios (RR) reported for the association of chronic kidney disease (as defined by estimated glomerular filtration rate <60 mL/min per 1.73 m^2^) and stroke risk using paired study estimates only. Risk ratios were adjusted for traditional cardiovascular risk factors (exact methods varied between studies).

Significant heterogeneity existed between multivariate-adjusted estimates among patients with an eGFR <60 mL/min per 1.73 m^2^ (*P*<0.001, *I*^2^=78.5%). The size of the estimate was reduced in a fixed-effects model but similar when the analysis was confined to studies that provided both unadjusted and adjusted risk estimates (Figure 2 and Materials and Appendix Figure III in the online-only Data Supplement). There was a similar association with ischemic (adjusted RR=1.25; 1.06–1.44) and hemorrhagic (adjusted RR=1.33; 0.97–1.82) stroke risk (Data and Appendix Figures IV and V in the online-only Data Supplement).

An eGFR <60 mL/min per 1.73 m^2^ was associated with an increased risk of subsequent stroke in all subgroups when estimates were stratified by study design, location, size, quality, duration of follow-up, GFR formula used, mean age groups, sex, race, percentage of diabetes mellitus/hypertensives/atrial fibrillation/smokers, setting of high-risk procedure, and stroke type (Table). These subgroups were investigated for heterogeneity, and although not statistically significant, there was a stronger association between CKD and stroke risk reported in smaller studies (<2500 participants; RR=1.73; 1.46–2.01), those with longer follow-up (≥96 months; RR=1.49; 1.31–1.70), when the Cockcroft and Gault GFR estimating equation was used (RR=1.55; 1.34–1.79), when the study quality was poor (RR=5.29; 2.25–12.40), when patients were younger (<60 years; RR=1.57; 1.38–1.77), when studies included a larger proportion (≥30%) of smokers (RR=1.52; 1.32–1.76), or when the event type reported were all recurrent (RR=1.65; 1.18–2.32). In addition, in studies where the participants were undergoing cardiac or carotid procedures, the excess risk of stroke was much greater (RR=1.73 [1.41–2.12] versus 1.33 [1.26–1.40] and 2.2 [0.92–5.26] versus 1.36 [1.29–1.43], respectively). The funnel plot also showed some asymmetry consistent with publication bias with smaller studies showing an exaggerated stroke risk in CKD (Materials and Appendix Figure VI in the online-only Data Supplement). Egger test confirms the presence of small-study effects in adjusted analysis (*P*=0.001).

**Table 1.**
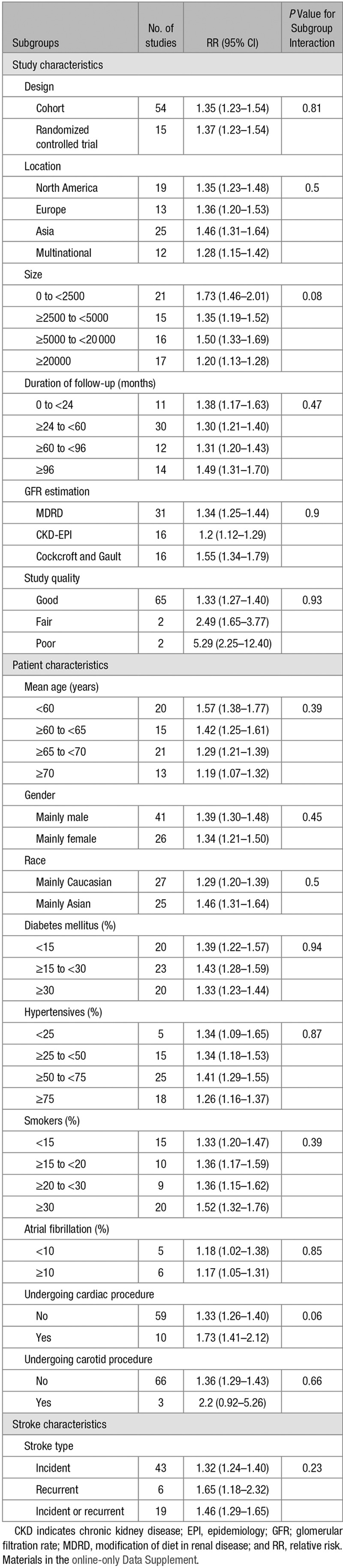
Subgroup Analysis and Meta-Regression: the Effect of Study, Participant and Stroke Characteristics on the Association Between CKD and Adjusted Risk of Stroke

**Figure 3.**
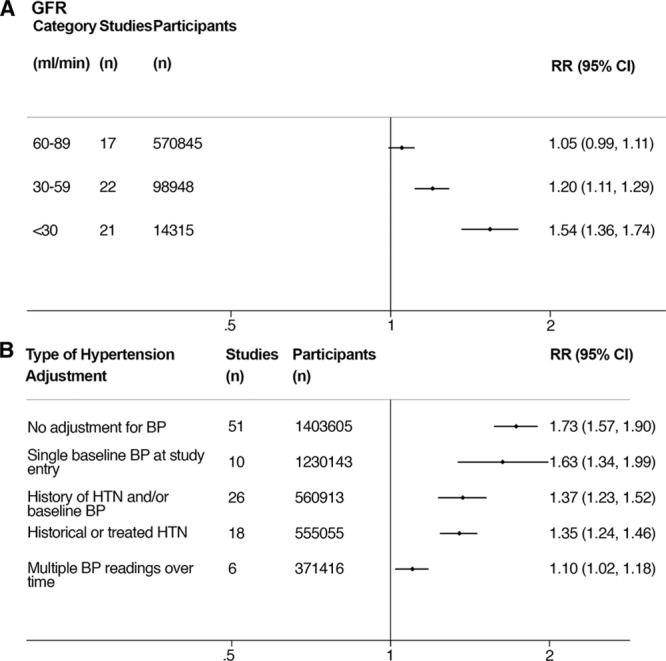
**A**, Adjusted relative risk (RR) of stroke by glomerular filtration rate (GFR) categories. **B**, Variation in the RR for the association of chronic kidney disease (CKD) and stroke risk depending on the method of hypertension adjustment used in the studies. All studies were also adjusted for other traditional risk factors. BP indicates blood pressure; and HTN, hypertension.

However, 2 meta-regression did reveal statistically significant heterogeneity. First, reported CKD severity (as defined by eGFR category) appeared to account for some of the heterogeneity. There was a nonsignificant 5% increased risk of stroke in participants with a GFR 60 to 89 mL/min per 1.73 m^2^ (adjusted RR=1.05; 0.99–1.11). The risk of stroke was increased by 20% in participants with a GFR of 30 to 59 mL/min per 1.73 m^2^ (adjusted RR=1.20; 1.11–1.29), rising to 54% in those with a GFR of <30 mL/min per 1.73 m^2^ (adjusted RR=1.54; 1.36–1.74; *P* trend <0.001; see Figure 3A).

Second, based on our proposed hierarchy of how hypertension was adjusted for in the included studies (Materials and Appendix in the online-only Data Supplement), Figure 3B demonstrates how the risk association between CKD and stroke varied depending on the method of hypertension adjustment employed in the studies, with progressive attenuation of risk with more complete adjustment (*P* for heterogeneity =0.004; adjusted *R*^2^=13.7%). The risk estimate when only studies adjusting for multiple prior blood pressure readings are included was only 1.10 (1.02–1.18; *I*^2^=31.6%, *P*_het_=0.2). Adjusting for only historical or treated hypertension appeared to have a similar effect to a more composite definition with minimal impact on the risk estimate when adjusting for single (or few) blood pressure readings at study or trial entry.

## Discussion

In this meta-analysis of 85 studies, which included 3 417 098 participants experiencing nearly 73 000 stroke events, we showed that patients with CKD (eGFR <60 mL/min per 1.73 m^2^) had a risk of stroke that was 36% greater than those without in multivariate-adjusted analysis. There was a dose-response relationship with the excess risk rising to 54% in those with advanced CKD (eGFR <30 mL/min 1.73 m^2^). These findings are consistent with stroke risk estimates from earlier, smaller meta-analyses.^10,11^ The risk association was similar for ischemic and hemorrhagic stroke subtypes (albeit insignificant in the latter) though this analysis was limited by lack of data reported on specific stroke subtypes in the majority of studies.

We used subgroup analyses to assess the influence of several factors on the association between CKD and stroke risk. The magnitude of risk was larger in smaller or poor-quality studies, in those with longer follow-up, in younger populations, in cardiac procedure-based studies, and in those that reported recurrent event types. The association of CKD and cerebrovascular disease (in the form of small vessel disease) has been previously shown to attenuate with adjustment for shared risk factors at older ages, but remained at younger ages, suggestive of a shared susceptibility to premature vascular disease.^17^ Similar to the earlier meta-analyses,^10,11^ we also found a higher stroke risk in Asian populations compared with white ones. This may be due to their higher prevalence of uncontrolled hypertension that tends to develop at an earlier age than other races.^18^

We report a hierarchy of adjustment for hypertension. The differential risk association between CKD and stroke depending on the method of hypertension adjustment used, particularly the effect of adjusting for multiple prior blood pressure readings, may have broader epidemiological implications for how we interpret allegedly independent relationships when the exact method of adjustment for confounding variables may be important. Based on these results, long-term blood pressure burden or control does appear to be a potentially important confounder of the CKD and stroke risk association. The degree of risk attenuation would tend to refute the hypothesis that low eGFR is a significant independent risk factor for incident stroke, outside of traditional vascular risk factors, particularly hypertension.

Adjusting for actual achieved blood pressure over time better reflects blood pressure control and duration, and may be a more meaningful method of considering this important confounder in the relationship between CKD and stroke than a single measurement or diagnostic label. A recently published post hoc analysis of the China Stroke Primary Prevention Trial aimed to test the impact of achieved blood pressure on first stroke and renal function decline among hypertensive patients with mild-to-moderate CKD.^19^ In patients with a time-averaged systolic blood pressure of ≤135 mm Hg compared with participants with a time-averaged on-treatment systolic blood pressure of 135 to 140 mm Hg, the incidence of total first stroke (1.7% versus 3.3%; hazard ratio, 0.51; 95% CI, 0.26–0.99) and ischemic stroke (1.3% versus 2.8%; hazard ratio, 0.46; 95% CI, 0.22–0.98) diminished significantly.

Understanding the long-term sequelae of middle life hypertension beyond the typical duration of BP trials is important as clinical practice shifts toward more intensive blood pressure control.^20^ This is particularly true for patients with CKD where the optimal blood pressure target is controversial and for whom most of the evidence comes from short trials insufficiently powered to assess cardiovascular outcomes.^21,22^ There have been concerns from SPRINT (Systolic Blood Pressure Intervention Trial)^20^ and other trials^21,23^ that treating to lower BP targets results in higher risk for acute kidney injury and more rapid loss of eGFR. However, in a SPRINT subgroup analysis of patients with CKD, those assigned to the intensive systolic blood pressure arm did not experience an increase in urinary biomarkers of tubule cell damage despite loss of eGFR.^24^ It is likely that eGFR declines in this setting reflect hemodynamic changes rather than intrinsic kidney cell damage in patients with CKD.

To the best of our knowledge, this study is the largest systematic review of CKD and stroke risk and the first to attempt to address confounding by hypertension in detail. However, this meta-analysis had several limitations. First, there was only one reviewer, and thus, the results may be biased if the selection criteria for including a study were applied in a subjective manner. To mitigate this risk, any questionable studies for inclusion were discussed with the senior author (Dr Rothwell). Second, a significant amount of heterogeneity was observed in the analysis and sub-analyses did not fully explain the variance although differential risk association depending on the type of hypertension adjustment did appear to account for a large part with very little heterogeneity in the subgroup with the most robust BP adjustment (*I*^2^=31.6%; *P*_het_=0.2). In addition, there was less statistical heterogeneity in studies of older (≥70 years) patients (RR=1.19, 1.07–1.32; *I*^2^=40.7%; *P*_het_=0.062) where the association between CKD and stroke is more likely to be confounded by traditional cardiovascular risk factors such as BP. There may be residual confounding in our results as we were unable to adjust for other known renal vascular risk factors, which may play a more important role in stroke causation in younger patients with CKD, accounting for the greater heterogeneity observed in this subgroup (*I*^2^=70.2%, *P*_het_<0.001). In a recent systematic review and meta-analysis of cardiovascular risk factors in CKD, serum albumin, phosphate, urate, and hemoglobin were all found to be statistically significant in their association with future cardiovascular events in addition to traditional risk factors.^25^ Third, since included studies employed a variety of GFR estimating equations, there was a risk of misclassification bias since the MDRD and the Cockcroft-Gault equations under- and over-estimate GFR, respectively. The adjusted stroke risk was lowest in the CKD-EPI studies (RR=1.20) which is known to correlate better with worse outcomes.^15^ Although albuminuria has been independently associated with a higher risk of stroke,^26^ we did not examine the interaction between eGFR and albuminuria. Finally, very few studies categorized stroke subtypes according to established stroke classification systems such as the modified TOAST (Trial of ORG 10172 in Acute Stroke Treatment) criteria.^27^ Stroke is a heterogeneous disease with different subtypes often reflecting different causes or mechanisms^28,29^ which may limit our ability to interpret a summary risk estimate for all-cause stroke in the CKD population.

Our meta-analysis found a significant association between CKD and increased stroke risk across various populations. However, the degree of attenuation of this risk with adjustment for traditional cardiovascular risk factors and then multiple prior blood pressure readings would question previous assertions of an independent relationship. To better understand the epidemiology of stroke risk in CKD, further studies should comprehensively explore the impact of adjustment for long-term blood pressure burden.

## Sources of Funding

Prof Rothwell has received funding from Wellcome Trust, Wolfson Foundation, British Heart Foundation, and the National Institute for Health Research Oxford Biomedical Research Center. Dr Kelly has received a scholarship from the Irish Nephrology Society.

## Disclosures

None.

## Supplementary Material
