# Supplementary material for: Does Chronic Kidney Disease Predict Stroke Risk Independent of Blood Pressure? A Systematic Review and Meta-Regression
Source: Stroke. 2019 Oct 9;50(11):3085–92. doi: 10.1161/STROKEAHA.119.025442 (PMC6824504; doi:10.1161/STROKEAHA.119.025442)
Supplement: Supplementary file 1 [file str-50-3085-s001.pdf]

SUPPLEMENTAL MATERIAL

Does chronic kidney disease predict stroke risk independent of blood pressure? A systematic review and meta-regression

Abbreviations:

AAA, abdominal aortic aneurysm; ACE, Angiotensin Converting Enzyme inhibitor; ACR, albumin:creatinine ratio; AF, atrial fibrillation; AR, aortic regurgitation; A2RB, Angiotensin 2 Receptor Blocker; BMI, body mass index; BMS, bare metal stent; Ca, calcium; CABG, coronary artery bypass grafting; CAD, coronary artery disease; CCF, congestive cardiac failure; CEA, carotid endarterectomy; CKD, chronic kidney disease; CLD, chronic liver disease; COPD, chronic obstructive pulmonary disease; CRP, C Reactive Protein; CVA, cerebrovascular accident; CVD, cardiovascular disease; DAPT, dual anti-platelet therapy; DBP, diastolic blood pressure; DM, diabetes mellitus; ECG, electrocardiograph; ETOH, alcohol; GFR, glomerular filtration rate; Hb, haemoglobin; HDL, high density lipoprotein; HIV, Human Immunodeficiency Virus; HRT, hormone replacement therapy; HTN, hypertension; IHD, ischemic heart disease; IS, ischemic stroke; LAD, left anterior descending artery; LDL, low density lipoprotein; LVEF, left ventricular ejection fraction; LVH, left ventricular hypertrophy; MDRD, Modification of Diet in Renal Disease; MI, myocardial infarction; NIHSS, National Institute of Health Stroke Scale; NSAIDs, Nonsteroidal anti-inflammatory drugs; NYHA, New York Heart Association; PCI, percutaneous coronary intervention; PCR, protein:creatinine ratio; PP, pulse pressure; PVD, peripheral vascular disease; Rx, treatment; SBP, systolic blood pressure; SD, standard deviation; SES, socio-economic status; STS score, Society of Thoracic Surgery score; TAVI, transcatheter aortic valve implantation; TC, total cholesterol; TE, thromboembolic; TG, triglyceride; TIA, transient ischemic attack.

Tables:

Appendix Table I. Search strategies

Appendix Table II. Characteristics of included studies

Appendix Table III. Characteristics of studies included in the meta-analysis

Appendix Table IV. Studies categorized according to a hierarchy of hypertension adjustment, from least (1) to best (4) adjustment

Supplemental Figures:

Appendix Figure I: Unadjusted risk ratio (RR) for the association of CKD (defined as eGFR < 60 ml/min/1.73m^2) and stroke risk

Appendix Figure II: Risk ratio (RR) for the association of CKD (defined as eGFR < 60 ml/min/1.73m^2) and stroke risk adjusted for traditional cardiovascular risk factors

Appendix Figure III: Risk ratio (RR) for the association of CKD (defined as eGFR < 60 ml/min/1.73m^2) and stroke risk adjusted for traditional cardiovascular risk factors, using a fixed-effects model

Appendix Figure IV: Risk ratio (RR) for the association of CKD and ischemic stroke risk adjusted for traditional cardiovascular risk factors

Appendix Figure V: Risk ratio (RR) for the association of CKD and hemorrhagic stroke risk adjusted for traditional cardiovascular risk factors

Appendix Figure VI: Funnel plot to assess for publication bias

Appendix Table I. Search strategies

MEDLINE	Embase
1. Kidney Diseases/	1. kidney disease/
2. exp Renal Replacement Therapy/	2. exp renal replacement therapy/
3. Renal Insufficiency/	3. dialysis.tw.
4. exp Renal Insufficiency, Chronic/	4. (CKF or CKD or CRF or CRD).tw.
5. dialysis.tw.	5. (end?stage kidney or end?stage renal).tw.
6. (hemodialysis or haemodialysis).tw.	6. (chronic kidney or chronic renal).tw.
7. (hemofiltration or haemofiltration).tw.	7. exp cerebrovascular accident/
8. (hemodiafiltration or haemodiafiltration).tw.	8. brain hemorrhage/
9. (end-stage renal or end-stage kidney or endstage renal or endstage kidney).tw.	9. brain infarction/
10. (ESRF or ESKF or ESRD or ESKD).tw.	10. stroke.tw.
11. (chronic kidney or chronic renal).tw.	11. exp proteinuria/
12. (CKF or CKD or CRF or CRD).tw.	12. albuminuria/
13. (CAPD or CCPD or APD).tw.	13. (proteinuria or ?albuminuria).tw.
14. (predialysis or pre-dialysis).tw.	14. or/1-6
15. or/1-14	15. or/7-10
16. exp Stroke/	16. or/11-13
17. Brain ischemia/	17. or/14, 16
18. Cerebral Small Vessel Diseases/	18. and/15, 17
19. Intracranial Hemorrhages/	
20. stroke.tw.	
21. (CVA or TIA).tw.	
22. or/16-21	
23. and/15,22	
24. albuminuria/	
25. proteinuria/	
26. (proteinuria or albuminuria).tw.	
27. or/24-26	
28. or/15,27	
29. and/22,28	

Appendix Table II. Characteristics of included studies

Study reference, Country, Name, (Reference)	Design, population, ethnicity	Size, (% men)	Mean or median age (SD or range)	GFR (ml/min/1.73m^2)	Albuminuria (category)	Stroke type(n) (Classification)	Follow-up (months)	Other stroke characteristics	Adjustment (Hypertension)
				**Formula** ***Reference*: range(n)** ***Comparison*: range (n)**	**Measurement** ***Reference*: range (n)** ***Comparison*: range (n)**				
Aguilar 2010, USA, Cardio-vascular Health Study, [1]	Cohort, No cerebrovascular disease, 14.4% DM, 61.8% HTN, 52.4% ever smoker, 3.9% AF. 17% African-American	3,205, (39)	78.5 (4.8)	MDRD *Reference*: ≥60 (unknown) *Comparison*: <60 (unknown)	ACR *Reference*: None (2,630) *Comparison*: Micro (560) Macro (15) Any (575)	Unspecified (26) Ischemic (316) Hemorrhagic (48)	104.4	Incident Fatal or non-fatal	Age, sex, race, BMI, smoking, hypertension, diabetes, LVH, AF, internal carotid artery stenosis ≥75%, SBP, DBP<br><br>(Categorical/Continuous variables - ≥140/90 or physician's diagnosis + use of Rx, SBP, DBP – average over 4-7 years)
Banerjee 2013, France, The Loire Valley Atrial Fibrillation Project [2]	Cohort 100% AF, 34.4% vascular disease, 16.4% DM, 12.6% smoking, 42.9% HTN. Unknown ethnicity	5912 (62.9)	70.1 (13.3)	MDRD *Reference:* ≥60 (2930) *Comparison:* 30-59 (2640) <30 (341)		Ischemic (171)	29.4	Incident	Age, gender, type of AF, hypertension, DM, vascular disease, heart failure<br><br>(Categorical variable - hypertension)
Bansal 2016, USA, CRIC study, [3]	Cohort 95% HTN, 61.4% DM, 14.8% smoking, 46.3% atherosclerotic disease. 46% black, 33.8%, white, 16.7% hispanic, 3.4% other	1794 (54)	59.9 (11.3)	MDRD *Reference:* None *Comparison:* <30 (1794)		Ischemic (67)	56.9	Incident	Age, sex, race, site, smoking, BMI, DM, proteinuria, statins, BP componenets & no. of BP medication classes<br><br>(Ordinal/categorical - variable –SBP [>140/120-140/<120],

									DBP [>90/80-90/<80], PP [69-153/51-69/15-51] & no. of med classes)
Bautistia 2015, USA, [4]	Cohort<br>100% AF, 78.1% HTN, 34.9% DM, 25.4% vascular disease, 15.8% prior stroke.<br>50% Hispanic, 32.2% Black, 17.7% Other	524 (55.1)	71	MDRD<br>*Reference:*<br>>90 (79)<br>*Comparison:*<br>60-90 (212)<br>45-59 (92)<br>30-44 (43)<br>15-29 (32)<br><15 (33)		Ischemic (145)	12	Incident	Age, gender, CCF, DM, HTN, previous stroke, vascular disease<br><br>(Categorical variable – HTN = multiple SBP>160 mmHg)
Bax 2008, Netherlands, [5]	Cohort,<br>22% diabetics, 30% cerebrovascular disease, 54% other atherosclerotic disease, 50.6% HTN, 81.2% ever smoker,<br>Unknown ethnicity	3,216, (76)	60 (10.4)	MDRD<br>*Reference*:<br>≥90 (602)<br>*Comparison*:<br>60-90 (2,097)<br><60 (517)	ACR, not used to estimate association with stroke<br>*Reference*:<br>None (2,646)<br>*Comparison*:<br>Micro (570)	Unspecified (112)	39	Incident or recurrent<br>Fatal or non-fatal	Age, sex, BMI, hypertension, IHD, previous CVA, PVD, AAA, diabetes, smoking, ACE/Angiotensin II Receptor Blocker use<br><br>(Categorical variable SBP≥160 mmHg or a DBP ≥95 mmHg) or on Rx – single reading)
Bedimo 2011, USA, [6]	Cohort,<br>100% HIV positive on retro-viral medication, 13% diabetics, 38% HTN, 29% smokers<br>Unknown ethnicity	19,424, (98)	46	MDRD<br>*Reference*:<br>≥90 (unknown)<br>*Comparison*:<br>60-90 (unknown)<br><60 (1,554)		Unspecified (868)	47.2	Incident<br>Fatal or non-fatal	Age, hypercholesterolemia, HTN, T2DM, smoking<br><br>(Categorical variable - history of hypertension)

Bos 2007, Netherlands, [7]	Cohort,<br>10% diabetics, no cerebrovascular disease, 12% IHD, 13% HTN, 5% AF.<br>99% White	4,937, (39)	68.9 (57.3-78.8)	Cockroft-Gault<br>*Reference:*<br>≥60 (2,652)<br>*Comparison:*<br><60 (2,285)	Unspecified (204)<br>Ischemic (338)<br>Hemorrhagic (44)	122.4	Incident or recurrent<br>Fatal or non-fatal	Age, sex, systolic/diastolic blood pressure, antihypertensive drug use, LVH, diuretic use, smoking, DM, cholesterol, HDL, uric acid, CRP, IHD, antithrombotic and lipid lowering drug use<br><br>(Continuous/categorical variables – SBP, DBP, Rx – 2 readings at same visit)
Cea Soriano 2015, UK, [8]	Cohort<br>100% T2DM, 56.5% HTN, 17.5% smoking, 9.8% previous stroke.<br>Unknown ethnicity	57946 (55.4)	65.7	MDRD<br>*Reference:*<br>≥60 (40034)<br>*Comparison:*<br>45-59 (12614)<br>30 –44 (4326)<br>15-29 (972)	Ischemic (3785)	81.1	Incident	Age, sex, BMI, smoking, HTN, hyperlipidaemia, history of MI/IS/TIA, IHD, duration of DM, HbA1c, polypharmacy<br><br>(Categorical variable - HTN)
Cheng 2008, Taiwan, [9]	Cohort,<br>9% diabetics, no vascular disease, 38.5% HTN, 23.1% current smokers,<br>Unknown ethnicity	17,026, (76)	57.2 (5.2)	MDRD<br>*Reference:*<br>≥90 (4190)<br>*Comparison:*<br>60-90 (11,583)<br><60 (1,253)	Unspecified (67)<br>Ischemic (28)<br>Hemorrhagic (57)	180	Incident or recurrent<br>Fatal	Age, sex, BMI, smoking, total cholesterol, hemoglobin, diabetes, systolic blood pressure, hypertension, cardiovascular disease<br><br>(Continuous/categorical variables – Hx of, >140/90, on Rx)

Codner 2016, Multinational, [10]	Cohort<br>100% TAVI patients, 95.1% HTN, 31.7% DM, 16.7% smoking, 14.4% previous MI.<br>Unknown ethnicity	1204 (44.5)	81.5 (5.7)	MDRD<br>*Reference:*<br>>60 (288)<br>*Comparison:*<br>31-60 (458)<br>≤30 (452)<br>15D (66)		Unspecified	12	Incident	Gender, device, NYHA class, STS score, peak gradient across the valve, LVEF<br><br>(Not adjusted for HTN)
Crimi 2016, Italy, PRODIGY trial, [11]	RCT<br>*Inclusion criteria:*<br>CAD requiring PCI<br>*Intervention:*<br>BMS, paclitaxel, zotarolimus-, or everolimus eluting stent.<br>6 vs 24mth DAPT<br>71.8% HTN, 24.4% DM, 24.2% smoking.<br>Unknown ethnicity	1981 (76.5)	75 [70-81]	MDRD<br>*Reference:*<br>≥ 60 (1608)<br>*Comparison:*<br><60 (373)		Unspecified (48)	24	Incident	Age, LVEF, DM, ACS at presentation, total stent length, DAPT duration, stent type.<br><br>(Not adjusted for HTN)
D'Ascenzo 2013, Italy, [12]	Cohort<br>100% TAVI patients<br>72.8% HTN, 28% DM, 23% cerebrovascular disease, 14.6% previous MI.<br>Unknown ethnicity	364 (34.5)	82.4 (5)	Cockcroft & Gault<br>*Reference:*<br>≥60 (72)<br>*Comparison:*<br>30-59 (219)<br>15-29 (73)		Unspecified (20)	18	Incident	Age, EF, pulmonary HTN, previous MI, PCI/CABG, PVD, previous stroke.<br><br>(Not adjusted for HTN)
De Leeuw 2002, Multinational, Syst-Eur trial, [13]	RCT,<br>*Inclusion criteria*: isolated systolic hypertension, age≥60.<br>*Intervention*: Ca-channel blocker +/- ACE<br>*Control:* Placebo<br>11% diabetics, 30% previous cardiovascular disease, 7.3% smoking<br>Unknown ethnicity	4,658, (33)	70 (6.6)	Cockcroft & Gault<br>Serum Creatinine Per 20 µmol/l increase	Dipstick<br>*Reference*:<br>None (4,225)<br>*Comparison*:<br>Micro (324)<br>Macro (109)	Unspecified (129)	24	Incident<br>Fatal or non-fatal	Active treatment, sex, age, systolic blood pressure smoking, previous cardiovascular disease, diabetes<br><br>(Continuous/categoric al variables – on Rx, SBP – 6 readings in 1-month run-in)

De Mattos 2006, USA, [14]	Cohort, 100% renal transplant recipients, 28% diabetics, 35% smokers, 56.2% HTN, 85% white, 4% black, 6% Asians, 4% Hispanic	922 (56)	44.2 (12.1)	Serum Creatinine; Reference: ≤140 µmol/l, eGFR >45 (593)<br><br>Comparison: >140 µmol/l, eGFR <45 (264)	Unspecified (48)	85	Incident or recurrent Fatal or non-fatal	Diabetes, age, prior CVA, peritoneal dialysis, BMI, era of transplant, pulse pressure.<br><br>(Continuous variable – PP)
Devbhandari 2006, UK, [15]	Cohort, 17% diabetics, 8% cerebrovascular disease, 76% known IHD, 53% hypertensive, 12.2% current smokers. Unknown ethnicity	19,558, (79)	63.4 (57-73)	Serum Creatinine *Reference*: <200 µmol/l, eGFR>60 (19,172) *Comparison*: >200 µmmol/l, eGFR <60 (386)	Unspecified (238)	36	Unknown	Propensity score: (Logistic EUROSCORE), hypertension, emergent procedure, cerebrovascular disease, diabetes, prior cardiac surgery, sex, EF<30%, BMI, respiratory disease, off-pump surgery, left main stenosis, hypercholesterolemia.<br><br>(Categorical variable - HTN = A history of blood pressure > 140/90 mm Hg or lower if treated)
Deo 2008, USA , Health ABC study, [16]	Cohort, 15% diabetics, 2% cerebrovascular disease, 9% IHD, 63.3% HTN. 10.3% smokers. 42% black	3,044, (49)	73.6 (2.8)	MDRD *Reference*: ≥60 (2389) *Comparison*: <60 (654)	Unspecified (163)	72	Incident Non-fatal	Race, age, sex, BMI, ETOH, smoking, diabetes, hypertension, aspirin, diuretic, ACE, beta-blocker, statin, LDL/HDL cholesterol, CRP, albumin, IL-6<br><br>(Categorical variable - HTN =self-report plus use of antihypertensive medications, or

								measured systolic blood pressure ≥140 mm Hg or diastolic blood pressure ≥ 90 mm Hg)
Dong 2017, China, [17]	Cohort<br>100% acute ischemic stroke. 67% HTN, 52.2% smoking, 40.2% DM, 10% CAD	972 (53.2)	68 (10.1)	MDRD<br>*Reference:*<br>≥90 (556)<br>*Comparison:*<br>60-90 (286)<br>30-59 (90)	Ischemic (79)	3	Recurrent	Unknown
Dukkipati 2004, USA, [18]	Cohort,<br>27% diabetics, 9% cerebrovascular disease, 100% undergoing percutaneous coronary intervention, 65% HTN. Unknown ethnicity	20,679, (68)	65 (12)	Cockroft Gault<br>*Reference*:<br>≥40 (unknown)<br>*Comparison*:<br><40 (unknown)	Unspecified (92)	Unknown	Incident and recurrent<br>Fatal and non-fatal	Age, sex, body surface area, diabetes, hypertension, hypercholesterolemia, CCF, previous PCI, PVD, emergent PCI, heparin prior to PCI, year, fluoroscopy time.<br><br>(Categorical variable - History of hypertension)
Dumonteil 2013, Multinational, PRAGMATIC-Plus Initiative study, [19]	Cohort<br>100% TAVI patients, 69.5% HTN, 45.2% CAD, 28.5% DM, 15.7% previous stroke	942 (53.8)	81 (7)	MDRD<br>*Reference:*<br>≥90 (109)<br>*Comparison:*<br>60-89 (329)<br>30-59 (399)<br><30 (72)<br>15D (33)	Major (22)<br>Minor (3)	1	Incident	Age, sex, DM, COPD, CAD, PVD, LVEF≤35, baseline anaemia category, learning effect, sheath size, access type, paravalvular AR grade ≥2.<br><br>(Not adjusted for HTN)

Ferro 2015,<br>UK, [20]	Cohort<br>100% TAVI patients<br>22.5% previous MI,<br>22.4% DM, 17.9%<br>previous stroke, 2.5%<br>smoking.<br>Unknown ethnicity	3696 (53.5)		MDRD<br>*Reference:*<br>≥60 (1390)<br>*Comparison:*<br>45-59 (1046)<br>30-44 (846)<br>15-29 (315)<br><15 or D (99)	Unspecified (96)	17.5	Incident	Sex, DM, COPD, extra cardiac arteriopathy, AF, previous cardiac surgery, BMI, LVEF<30%, no coronary vessel with >50% stenosis, procedure urgency, aortic valve gradient, non-transfemoral approach, successful valve deployment.<br><br>(Not adjusted for HTN)
Ford 2009,<br>Multinational,<br>PROSPER<br>study, [21]	RCT,<br>*Inclusion criteria*: Pre-existing or increased risk of vascular disease.<br>*Intervention*: Pravastatin<br>*Control*: Placebo,<br>12.8% DM, 61.9%<br>HTN, 26.9% current<br>smokers.<br>Unknown ethnicity	5,796,<br>(48)	75.3 (3.3)	MDRD<br>*Reference:*<br>≥60 (2,702)<br>*Comparison*:<br>50-60 (1,641)<br>40-50 (1,104)<br>20-40 (349)	Unspecified (415)	38	Incident or<br>recurrent<br>Fatal or non-fatal	Randomized treatment, country, sex, smoking, age, hypertension, diabetes, previous vascular disease, systolic/diastolic blood pressure, LDL, HDL, glucose, BMI, CRP.<br><br>(Continuous/categorical variables – HTN, SBP/DBP)
Garcia—<br>Carretero 2017,<br>Spain, [22]	Cohort<br>100% HTN, 30.6% DM,<br>Unknown ethnicity	2016 (49.3)	57 (17)	CKD-Epi<br>*Reference:*<br>>90 (1198)<br>*Comparison:*<br>60-90 (630)<br>≤ 60 (188)	Unspecified (52)	55.2	Incident	Age, gender, DM, HDL-C, LDL-C.<br><br>(Not adjusted for HTN)

Garcia-Gill 2016, Spain, [23]	Cohort<br>21% HTN, 34.3% smoking.<br>Unknown ethnicity	1,081,865 (49.1)	49.5 (11.6)	CKD-Epi<br>*Reference:*<br>≥90 (703242)<br>*Comparison:*<br>60-89 (354041)<br>45-59 (20465)<br>30-44 (3447)<br>15-29 (475)<br><15 (195)		Unspecified (8900)	60	Incident	Age, sex, smoking, DM, SBP, DBP, TC, HDL-C.<br><br>(Continuous variable)
Gelsomino 2017, Italy, [24]	Cohort<br>100% CABG patients, 64.3% HTN, 18.3% DM, 0.6% CVD.<br>Unknown ethnicity	1186 (84.9)	69.1 (8.7)	CKD-Epi<br>*Reference:*<br>≥60 (791)<br>*Comparison:*<br>45-59 (224)<br>30-44 (93)<br>≤29 (78)		Unspecified<br>30d -early (124)<br>late (72)	66	Incident	Age, LVEF.<br><br>(Not adjusted for HTN)
Go 2009, USA, [25]	Cohort,<br>17% diabetics, 9% cerebrovascular disease, 59% other vascular disease.,50.9% HTN, 100% AF.<br>86% White, 4% black, 5% Asian	13,535, (57)	71.6 (unknown)	MDRD<br>*Reference*:<br>≥60 (13,535)<br>*Comparison:*<br>45-60 (7,746)<br><45 (5,789)	Dipstick<br>*Reference*:<br>None (unknown)<br>*Comparison*:<br>Macro (unknown)	Ischemic (637)	96	Incident or recurrent<br>Fatal or non-fatal	Age, sex, race, SES, educational attainment, prior ischemic stroke, CCF, diabetes, hypertension, IHD<br><br>(Categorical variable - Hypertension identified from outpatient sources.)
Gruberg 2014, USA, CARE Registry, [26]	Cohort<br>100% CAS or CEA, 89.1% HTN, 73.8% smoker, 53.8% IHD, 35.9% DM, 45% prior stroke.<br>93.5% White	11832 (60.3)	70.8 (10.5)	MDRD<br>*Reference:*<br>≥90 (2042)<br>*Comparison:*<br>60-89 (5359)<br>30-59 (4116)<br>≤29 (315)		Unspecified<br>In-hospital (353)<br>30-day (461)	1	Incident	Age, HTN, future major surgery, prior stroke, symptomatic lesions, CCF.<br><br>(Categorical variable)

Guo 2013, China, [27]	Cohort,<br>100% AF. 78% HTN, 46% diabetics, 68% IHD, 19% PVD, 25% known cerebrovascular disease	617 (unknown)	78 (11)	MDRD<br>*Reference*:<br>≥60 (541)<br>*Comparison:*<br><60 (76)	Ischemic (40)	12	Incident or recurrent<br>Fatal or non-fatal	Age, sex, HTN, diabetes, CCF, vascular disease, prior stroke, warfarin/statin/diuretic use.<br><br>(Categorical variable - HTN: resting blood pressure ≥ 140 mm Hg systolic and/or ≥ 90 mm Hg diastolic on at least 2 occasions or current antihypertensive drug treatment.)
Holme 2010, Multinational, IDEAL trial, [28]	RCT,<br>*Inclusion criteria*: ≥80 years, or younger and previous myocardial infarction.<br>*Intervention*: Simvastatin 20mg<br>*Control*: Atorvastatin 80mg<br>12% diabetics, 7% known cerebrovascular disease, 40% previous coronary revascularization, 32.9% HTN, 79.1% ever smokers, 7.5% AF.<br>Unknown ethnicity	8,863, (unknown)	61.8 (9.5)	MDRD<br>*Reference*:<br>≥60 (6,542)<br>*Comparison*:<br><60 (2,321)	Unspecified (323)	57.6	Incident or recurrent<br>Fatal or non-fatal	CCF at baseline.<br><br>(Not adjusted for hypertension)

Holzmann 2012,<br>Sweden,<br>AMORIS study, [29]	Cohort,<br>3% DM	539,287,<br>(53.3)	47.7 (14.2)	MDRD<br>*Reference:*<br>>90 (477,046)<br>*Comparison*:<br>60-90 (59,016)<br>30-60 (3,006)<br>15-30 (350)		Unspecified (467)<br>Ischemic (2,284)<br>Hemorrhagic (605)	141.6	Incident<br>Fatal or non-fatal	Age, sex, diabetes, total cholesterol, triglycerides.<br><br>(Not adjusted for hypertension)
Hwang 2014, South Korea, [30]	Cohort<br>100% AMI, 50% HTN, 42.7% smoking, 31.5% DM, 4.2% AF	4738 (71.5)	62.6 (12)	CKD-Epi<br>*Reference:*<br>≥60 (3556)<br>*Comparison:*<br><60 (1182)		Unspecified (84)	42	Incident	Age, gender, HTN, DM, smoking, stroke history, Kilip class, LVEF, Hb, hsCRP, medical treatment at discharge.<br><br>(Categorical variable)
Irie 2006,<br>Japan, [31]	Cohort,<br>7% diabetics,<br>Mainly Asian	91,432,<br>(34)	58.8<br>(unknown)	MDRD<br>*Reference*:<br>≥100 (17,636)<br>*Comparison*:<br>90-100(21,846)<br>80-90 (20,402)<br>70-80 (20,461)<br>60-70 (8,190)<br><60 (2,897)	Dipstick<br>*Reference*:<br>None (88,438)<br>*Comparison*:<br>Macro (1,929)	Unspecified (985)	120	Incident<br>Fatal	Age, hypertension, smoking, ETOH, diabetes, total cholesterol, HDL cholesterol, BMI, urinary protein (for eGFR analyses)<br><br>(Categorical variable – adjusted for hypertensive category).
Itaya 2010,<br>Japan, [32]	Cohort,<br>51% diabetics, 100% undergoing percutaneous coronary intervention for evaluation of chest pain, 29.6% HTN, 45.3% smoking,<br>Unknown ethnicity	715,<br>(74)	66.8<br>(11.6)	MDRD<br>*Reference*:<br>≥60 (563)<br>*Comparison*:<br><60 (152)		Unspecified (12)<br>Hemorrhagic (1)	20.9	Incident<br>Fatal or non-fatal	Unadjusted

Ix 2005, Multinational, ARTS study, [33]	RCT, *Inclusion criteria*: Ischemic cardiac symptoms *Intervention*: PCI *Control*: CABG, 18.1% DM, 50.4% HTN, 13.2% current smokers. Unknown ethnicity	1,205, (77)	60.5 (9.4)	Cockcroft Gault *Reference*: ≥60 (886) *Comparison*: <60 (290)	Unspecified (37)	36	Incident Fatal or non-fatal	Diabetes, hypertension, ejection fraction, ACE, aspirin, PVD, hemoglobin, COPD, silent ischemia, hyperlipidemia. (Categorical variable – HTN = blood pressure >160/95 on repeat measurements or on antihypertensive medications)
John 2000, USA, [34]	Cohort, 28% diabetics, 68.6% HTN, 7.5% smokers, 13% cerebrovascular disease, 31% IHD. 100% undergoing coronary revascularization, Unknown ethnicity	19,224, (72)	65 (10)	Serum Creatinine *Reference*: ≥2.5mg/dl, eGFR >30 (583) *Comparison*: <2.5mg/dl, eGFR<30 (18,641)	Unspecified (270)	0.03	Incident and recurrent Fatal or non-fatal	Previous CVA, smoking, carotid vascular disease, age, PVD, diabetes, previous CABG. (Categorical variable)
Kokubo 2009, Japan, [35]	Cohort, 5% diabetics, no previous vascular disease, 30.3% HTN, 29.9% current smokers. Mainly Asian	5,494, (47)	54.7 (13.2)	MDRD *Reference*: ≥90 (2,415) *Comparison*: 60-90 (2,452) 50-60 (387) <50 (124)	Unspecified (18) Ischemic (141) Hemorrhagic (54)	140.4	Incident Fatal or non-fatal	Age, sex, BMI, smoking, ETOH, hypertension, diabetes, hypercholesterolemia. (Ordinal variables - At the time of the baseline examination, subjects were classified into 1 of the 5 BP categories based on the European Society of Hypertension and European Society of Cardiology (ESH-ESC) 2007 criteria:

									optimal (SBP <120 mm Hg and DBP <80 mm Hg), normal (SBP 120 to 129 mm Hg or DBP 80 to 84 mm Hg), high-normal BP (SBP 130 to 139 mm Hg or DBP 85 to 89 mm Hg), and hypertensive (SBP ≥140 mm Hg or DBP ≥90 mm Hg).
Konishi 2011, Japan, [36]	Cohort,<br>100% undergoing CABG, 38% diabetics, 67% HTN, 69% smokers, 48% IHD, 4% known cerebrovascular disease, 12% AF	1,809 (85)	54.5 (12.0)	Japan-specific<br>*Reference*:<br>>60 (1,488)<br>*Comparison:*<br><60 (321)		All-cause (127)<br>Unspecified.	136.8	Incident or recurrent<br>Fatal or non-fatal	Age, sex, HTN, SBP/DBP, diabetes, previous IHD or CVA, re-vascularization, aspirin.<br><br>(Categorical/continuous variables - Hypertension was defined as a systolic blood pressure ≥140mmHg, a diastolic blood pressure ≥90 mmHg, or treatment with antihypertensive medications.)<br><br>.
Koolman 2014, Netherlands, [37]	Cohort<br>100% AF, 55.1% HTN, 17.5% prior stroke, 17% DM.<br>Unknown ethnicity	724 (56.5)	74.8 (9.7)	MDRD<br>*Reference:*<br>>60 (300)<br>*Comparison:*<br>30-60 (294)		Unspecified (45)	25.2	Incident	Age, gender, antiplatelets, NSAIDs, HTN, DM, CCF.<br><br>(Categorical variable)

				<30 (130)					
Koren-Morag 2006,<br>Israel,<br>BIP study, [38]	RCT,<br>*Inclusion criteria*: IHD<br>*Intervention*:<br>Bezafibrate<br>*Control*: Placebo<br>16% diabetics, no cerebrovascular disease, 100% other vascular disease, 31.8% HTN, 10.7% smokers.<br>Unknown ethnicity	6,685,<br>(89)	59.4 (7.1)	Cockcroft Gault<br>*Reference*:<br>≥60 (5,345)<br>*Comparison*:<br><60 (1,340)		Ischemic (287)	57.6 to 97.3	Incident or recurrent<br>Fatal or non-fatal	Age, sex, inclusion in clinical trial, systolic blood pressure, HTN, diabetes, TGs, %HDL, NYHA functional class, BMI, PVD, smoking, anti-platelets, anti-hypertensives, lipid-lowering agents.<br><br>(Categorical/continuous variables – HTN, SBP)
Kovesdy 2016, USA, RCAV Study, [39]	Cohort<br>87% HTN, 49% DM, 40% CAD.<br>79% White, 17% Black, 2% Hispanic, 2% Other	339887 (97)	69 (10)	CKD-Epi<br>*Reference:*<br>None<br>*Comparison:*<br><60 (339887)		Ischemic (14557)	57.6	Incident	Age, sex, race, marital status, income, HTN, DM, CAD, CCF, previous stroke, PVD, malignancy, liver disease, rheumatologic disease, chronic lung disease, dementia, HIV/ AIDS, depression, weight loss, number of antihypertensive, SBP or DBP.<br><br>(Categorical/continuous variables - baseline BP = average of all measurements during the first 90 days following cohort entry)

Kurth, 2009,<br>USA,<br>Women's health study, [40]	Cohort,<br>2% diabetics, no known vascular disease, 25.1% HTN. 11.7% current smokers.<br>94% White.	27,939,<br>(0)	54.7 (1.1)	MDRD<br>*Reference*:<br>$\geq$90 (14,979);<br>*Comparison*:<br>75-90 (8,073)<br>60-75 (3,572)<br><60 (1,315)		Unspecified (389)	144	Incident<br>Fatal or non-fatal	Age, SBP, HTN, antihypertensive treatment, smoking, body mass index, alcohol consumption exercise, total cholesterol concentration, C reactive protein concentration, postmenopausal hormone use, history of diabetes, as well as randomised treatment assignments.<br><br>(Categorical/continuous variables - History of hypertension (systolic blood pressure $\geq$140 mmHg, diastolic blood pressure $\geq$90 mm Hg, or use of antihypertensive drugs), SBP)

Kushiro 2017, Japan, HONEST Study, [41]	Cohort<br>100% HTN,<br>20.5% DM,<br>12.3% smoking,<br>10.5% CVD.	21591 (49.4)	64.9 (11.9)	MDRD<br>*Reference:*<br>≥60 (17082)<br>*Comparison:*<br><60 (4346)	Atherothrombotic infarction (42)<br>Cardioembolic (3)<br>Lacunar (40)<br>Unclassified (16)<br>ICH (17)<br>SAH (8)	24	Incident	Age, sex, DM, FHx of CVD, past CVD, smoking, dyslipidaemia, HTN type.<br><br>(Categorical variable in stratified analysis according to HTN – well controlled, white coat, masked, poorly controlled)
Kuwashiro 2012, Japan, Fukuoka stroke registry, [42]	Cohort,<br>35% diabetics, 52% smokers, 100% ischemic stroke, 76.5% HTN, 8.6% AF.<br>Unknown ethnicity	876<br>(61)	69.8 (11.5)	Japan-specific<br>*Reference:*<br>≥60 (641);<br>*Comparison:*<br><60 (235)	Ischemic (876)<br>(TOAST)	12	Incident<br>Non-fatal	Age, systolic blood pressure, anti-hypertensive treatment, smoking, BMI, ETOH, exercise, total cholesterol, CRP, HRT, diabetes, assigned treatments.<br><br>(Categorical/continuous variables – on Rx, SBP)
Lee 2013, Multinational, VISP trial, [43]	RCT,<br>*Inclusion criteria*: Prior ischemic stroke<br>*Intervention*: High dose vitamin B<br>*Control*: Low dose vitamin B<br>79% white, 15% black, 6% other. 74% HTN, 29% diabetics, 16% IHD, 17% smokers.	3,673 (63)	66.3 (10.8)	CKD-EPI<br>*Reference*:<br>60-74 (881)<br>*Comparison*:<br>>105 (220)<br>90-104 (663)<br>75-89 (942)<br>45-59 (631)<br><45 (336)	Ischemic (300)	20	Recurrent<br>Fatal or non-fatal	Age, sex, previous CEA, diabetes, IHD, SBP, CCF, smoking, ETOH, BMI, anti-thrombotic use, homocysteine level.<br><br>(Continuous variable - average seated systolic blood pressure during the trial (follow up at 6/12/24 mths))

Lee 2016, South Korea, [44]	Cohort<br>74.9% HTN, 28.1% DM, 25.4% smoking, 19.7% IHD	295 (53.2)	67.6 (14-94)	CKD-Epi<br>*Reference:*<br>≥60 (239)<br>*Comparison:*<br><60 (56)	UACR<br>*Reference:*<br><30 (165)<br>*Comparison:*<br>≥30 (130)	Ischemic (26)<br>SAH (1)	22	Recurrent<br>Fatal or non-fatal	Age, sex, DM, HTN, smoking, AF, previous stroke, alcohol Hx, NIHSS score.<br><br>(Categorical variable – on treatment or SBP≥140 or DBP≥90 on repeated exam)
Li 2015, China, [45]	Cohort<br>43.6% HTN, 32.8% smoking, 9% DM	92013 (70.6)	51.8	CKD-Epi<br>*Reference:*<br>≥90 (30609)<br>*Comparison:*<br>60-89 (49089)<br>30-60 (11801)<br><30 (514)	Urine dipstick<br>*Reference:*<br>None (88164)<br>*Comparison:*<br>≥1+ (3849)	Ischemic (1128)<br>Hemorrhagic (406)	48	Incident	Age, sex, smoking, drinking, BMI, LDL-C, HDL-C, TG, TC, DM, HTN, hyperlipidaemia, AF.<br><br>(Categorical variable - SBP≥140 or DBP≥90 or on Rx or self-reported Hx)
Luo 2014, China, China National Stroke Registry, [46]	Cohort<br>100% T2DM, 71.3% HTN, 36.2% smoking, 15.7% CAD	4836 (60.5)	64.9 (11.4)	CKD-Epi<br>*Reference:*<br>≥120 (387)<br>*Comparison:*<br>90-119 (2209)<br>60-89 (1591)<br>45-59 (381)<br><45 (268)		Unspecified (1012)	12	Recurrent<br>Fatal or non-fatal	Age, sex, history of stroke, HTN, hyperlipidaemia, AF, CAD, DM, smoking, alcohol, BMI, baseline NIHSS, lipid-lowering drug at discharge, antihypertensive drug at discharge, pneumonia, urethral infection.<br><br>(Categorical variable - SBP≥140 or DBP≥90 or on Rx or self-reported Hx)

Mann 2001, USA, HOPE trial, [47]	RCT, *Inclusion criteria*: Vascular disease or diabetes and another risk factor *Intervention:* Ramipril, Vitamin E *Control:* Placebo 42% diabetics, 7% known cerebrovascular disease, 80% IHD, 49.7% HTN, 71.1% ever smoker. Unknown ethnicity	9,287, (73)	65.9 (6.7)	Cockcroft Gault *Reference*: ≥65 (5,888) *Comparison*: <65 (3,394)	ACR, but not used to estimate association with stroke. All participants had < macro	Unspecified (381)	54	Incident Fatal or non-fatal	Age; sex; waist-to-hip ratio; body mass index; and history of hypertension, diabetes, coronary artery disease, peripheral vascular disease, smoking, ramipril use, renal insufficiency, SBP/DBP<br><br>(Both continuous and categorical variables – SBP, DBP, History of HTN)
Mann 2008, Multinational, HOPE-2 trial, [48]	RCT, *Inclusion criteria:* >55 years, high cardiovascular risk. *Intervention:* Folic acid, vitamins B6, B12. *Control:* Placebo 38% diabetics, 9% cerebrovascular disease, 85% other vascular disease, 53.5% HTN. 12% smokers. Unknown ethnicity	3,296, (75)	69 (6.7)	MDRD *Reference:* ≥60 (2,691) *Comparison*: <60 (619)		Unspecified (41)	60	Incident and recurrent Fatal or non-fatal	Gender, systolic and diastolic blood pressure, body mass index, waist-to-hip ratio, history of hypertension, history of stroke and active treatment.<br><br>(Both continuous and categorical variables – SBP, DBP, hx of)
Marui 2013, Japan, CREDO-Kyoto, [49]	Cohort, 100% PCI/CABG, 71.3% HTN, 43.2% DM, 25.4% smoking	1842 (75.1)	66.7 (8.4)	Cockcroft & Gault *Reference:* ≥60 (1339) *Comparison:* <60 (503)		Unspecified (144)	44.4	Incident	Age, sex, BMI, emergency procedure, prior MI, CCF, stroke, PVD, AF, COPD, malignancy, HTN, DM, hyperlipidaemia, anaemia, proximal LAD & use of certain vessels.<br><br>(Categorical variable)

Matsue 2013, Japan, [50]	Cohort<br>100% AMI with PCI, 60.9% smoking, 60.6% HTN	312 (77.6)	66.9 (11.2)	MDRD<br>*Reference:*<br>≥60 (146)<br>*Comparison:*<br><60 (166)		Ischemic	46.1	Incident	Age, sex, smoking, HTN, DM, Hyperlipidaemia, cerebral infarction, medications, culprit lesion, EF,<br>max CK, TC, LDL-C.<br><br>(Categorical variable = SBP ≥140 +/or DBP≥90 or on Rx)
McAlister 2017, Canada, [51]	Cohort<br>100% AF, 64.1% HTN, 21.6% DM, 11.3% CAD.<br>Unknown ethnicity	58451 (53.2)	66	CKD-Epi<br>*Reference:*<br>≥60 (44217)<br>*Comparison:*<br>45-59 (8046)<br>30-44 (4264)<br><30 (1924)	Urine dip/ACR/PCR<br>*Reference:*<br>Neg/<3/<15 (52132)<br>*Comparison:*<br>Trace or 1+/ 3-30/15-50 (3354)<br>2+/>30/>50 (2965)	Unspecified (5620)	31	Incident	Age, sex, aboriginal status, social assistance, postal code income quintile, rural/urban status, previous TE or bleeding event, CCF, HTN, DM, PVD.<br><br>(Categorical variable)
McMullan 2014, Multinational, Jichi Medical School ABPM Study, Miyazaki ABPM study, AASK cohort study, [52]	Pooled cohort<br>100% HTN, 17% CVD, 16% smoking.<br>50% Japanese,<br>50% African American	394 (46)	67.5 (6.7)	CKD-Epi<br>*Reference:*<br>≥65 (None)<br>*Comparison:*<br><65 (394)		Unspecified (22)		Incident	Age, gender, smoking, BMI, mean 24h SBP, LDL-C, glucose. HR for sleep trough morning blood pressure surge, (10 mmHg), nocturnal dipping (10% increments) & events.<br><br>(Continuous variables)

Micelli 2011, Multinational, [53]	Cohort,<br>15% diabetics, 100% undergoing coronary artery bypass grafting, 65.2% HTN.<br>Unknown ethnicity	9,159, (80)	64.1 (9.2)	Cockcroft Gault<br>*Reference*:<br>≥60 (5,484)<br>*Comparison*:<br><60 (3,675)		Unspecified (53)	1	Incident or recurrent<br>Fatal or non-fatal	Age, sex, functional capacity, diabetes, hypertension, COPD, neurologic disease, ejection fraction, recent MI, number of previous grafts, off-pump surgery, propensity score<br><br>(Categorical variable - History of hypertension)
Muntner 2012, USA , REGARDS study, [54]	Cohort,<br>13% smokers, 48% hypertensive, 17.9% diabetic.<br>37% black	20,386, (46)	64.4 (9.2)	CKD-EPI<br>*Reference*:<br>>90 (9,431)<br>*Comparison*:<br>60-90 (9,053)<br>45-60 (1,321)<br><45 (581)	ACR<br>*Reference*:<br>None (13,310)<br>*Comparison*:<br>Micro (6,844)<br>Macro (440)	Unspecified (2,548)	25.2	Incident<br>Fatal or non-fatal	Age, race, sex, geographic region, education, household income, smoking, ETOH, BMI, systolic blood pressure, antihypertensive medication use, dyslipidemia, diabetes and CRP<br><br>(Continuous & Categorical variables – SBP, on Rx)
Nagai 2014, Japan, [55]	Cohort<br>26.3% HTN, 13.7% smoking	298148 (39.7)	63.2 (8.1)	MDRD<br>*Reference:*<br>≥60 (98987)<br>*Comparison:*<br><60 (19391)	Urine dipstick<br>*Reference:*<br>Negative/trace (284567)<br>*Comparison:*<br>≥1+ (13581)	Unspecified (4426)	36	Incident	Age, sex, BMI, HTN category, smoking, anti-dyslipidaemia drugs, hyperglycemia, hypoglycemic drugs.<br><br>(Categorical variable – HTN categories = normotensive, untreated, treated, drug-resistant. SBP≥140 or DBP≥90 mmHg)

Nakagawa 2011, Japan, [56]	Cohort, 100% chronic or paroxysmal atrial fibrillation, 17% DM, 41% HTN Unknown ethnicity	387, (75)	66 (11)	Japan-specific *Reference*: >60 (258) *Comparison*: <60 (129)		Ischemic (5) Hemorrhagic (2)	67.2	Incident or recurrent Fatal or non-fatal	Not adjusted for hypertension.
Nakamura 2009, Japan, MEGA study, [57]	RCT, *Inclusion criteria*: Hypercholesterolemia, no previous cardiovascular events. *Intervention:* Pravastatin + dietary intervention *Control:* Dietary intervention alone. 20.9% DM, 43.1% HTN, 15% smokers. Unknown ethnicity	7,196, (unknown)	58.2	MDRD *Reference*: >60 (2,156) *Comparison*: 30-60 (1,516)		Ischemic (43) Hemorrhagic (13)	63.6	Incident or recurrent Fatal or non-fatal	Age, sex, baseline HDL-cholesterol, hypertension, diabetes, smoking status (Categorical variable)
Nakayama 2007, Japan, Okashama study, [58]	Cohort, 18.6% DM, 22.5% HTN, 15.6% ever smokers Mainly Asian	1,977, (37)	62.9 (	Cockcroft Gault *Reference*: >70 (555) *Comparison*: 40-70 (1,246) <40 (176)	Dipstick Reference: Micro (unknown) Comparison: Macro (unknown)	Unspecified (112)	96	Incident Fatal or non-fatal	Age, sex, systolic blood pressure, BMI, smoking, use of antihypertensive medication, history of cardiovascular disease, hypercholesterolemia and diabetes (Categorical and continuous variables – SBP, use of antihypertensive

								medications)
Nickolas 2008, USA, NOMAS study, [59]	Cohort, 19.6% DM, 47.3% ever smoker, 57% White, 22% black	3,298, (33)	69.2 (10.1)	Cockcroft Gault *Reference*: > 60 (945) *Comparison*: 15-60 (2,353)	Ischemic (177) Hemorrhagic (24)	78	Incident Fatal or non-fatal	Age, sex, education, hypertension, LDL cholesterol, DM, IHD, smoking, ETOH<br><br>(Categorical variable)
Ninomiya 2005, Japan, Hisayama study, [60]	Cohort, 10.9% DM, 37.9% HTN, 25.2% smokers. 100% Asian	2,634, (42)	59.4 (11.7)	MDRD *Reference*: >60 (2,364) *Comparison*: 15-60 (270)	Ischemic (137) Hemorrhagic (60)	144	Incident Fatal or non-fatal	Age, systolic blood pressure, antihypertensive medication, ECG abnormalities, diabetes, total cholesterol, HDL, TGs, BMI, smoking, ETOH, homocysteine, CRP<br><br>The mean of three measurements was used for the analysis. Hypertension was defined as blood pressure ≥140/90 mm Hg and/or current use of antihypertensive agents.<br><br>(Continuous and categorical variables - systolic blood pressure, antihypertensive medication, hypertension)

Ohsawa 2013, Japan, Iwate-KENCO study, [61]	Cohort<br>40.2% HTN, 24.1% smoking, 5.1% DM	24560 (34.1)	62.3 (11.4)	CKD-Epi *<br>*Reference:*<br>≥90 (2508)<br>*Comparison:*<br>60-90 (20612)<br>45-60 (1259)<br><45 (190)<br><br>*MDRD also reported	UACR<br>*Reference:*<br>None (18620)<br>*Comparison:*<br>30-299 (5453)<br>≥300 (487)	Unspecified (605)	67.2	Incident	Age, sex, SBP, BMI, TC, HDL-C, HbA1c, smoking habit, regular drinking habit, exercise habit.<br><br>(Continuous variable – SBP)
Papademetriou 2016, USA/Canada, ACCORD, [62]	RCT<br>*Inclusion criteria*<br>T2DM with HbA1c ≥ 7.5%, age 40-79 with CVD or 55-79 significant atherosclerosis, albuminuria, LVH, or other RFs><br>*Intervention:*<br>Target SBP < 120 mmHg<br>*Control:*<br>Target SBP < 140 mmHg<br>54.2% smoking, 33.7% CVD, 58.9% White, 23.7% Black, 6.8% Hispanic	4678 (52.3)	62.2 (6.9)	MDRD<br>*Reference:*<br>≥90 (3645)<br>*Comparison:*<br>60-89 (693)<br>30-59 (632)		Unspecified (95)	42	Incident<br>Fatal or non-fatal	Study group assignment, center, age, gender, previous CVD/CV event, BMI, HbA1c, SBP, smoking, insulin, anti-hypertensive use.<br><br>(Continuous & categorical variables - SBP, study group, on treatment)
Papademetriou 2017, Multinational, ORIGIN Trial, [63]	RCT<br>*Inclusion criteria:*<br>≥50 years with pre- or early T2DM with prior CV event or additional RFs.<br>*Intervention:*<br>Basal insulin<br>*Control:*<br>Standard care without insulin.	12174 (65)	63.5 (7.81)	MDRD<br>*Reference:*<br>≥90 (2940)<br>*Comparison:*<br>60-89 (6855)<br>30-59 (2379)	UACR<br>*Reference:*<br><30 (9786)<br>*Comparison:*<br>30-300 (1838)<br>>300 (550)	Unspecified (634)	74.4	Incident<br>Fatal or non-fatal	Age, gender, BMI, factorial allocation, glycemic status, CVD Hx, smoking, SBP, PP, HR, history of HTN, use of ACE/ARB or statins, fasting glucose, HbA1c, K+ & lipid levels.<br><br>(Continuous &

	88% T2DM, 12% Pre-diabetes, 79.4% HTN, 12.3% smoking.<br>59% White, 25.3% Latin, 3.3% Black							categorical variables - SBP at entry & Hx of HTN)
Patel 2017, USA, [64].	Cohort<br>100% PCI, 83.3% HTN, 37.7% DM, 28.6% smoking.<br>49.2% White, 14.9% Black, 11.2% Asian, 20.4% Hispanic	6478 (71.3)	67.1 (12.2)	CKD-Epi<br>*Reference:*<br>>90 (1351)<br>*Comparison:*<br>60-89 (2882)<br>30-59 (1742)<br>15-29 (191)<br><15 or D (312)	Unspecified (20)	42	Incident	Age, CCF NYHA class, previous stroke, HTN, EF <40%, history of PCI, statin, beta blocker, ACE/ARB use, post-procedure transfusion.<br><br>(Categorical variable)
Perticone 2009, Italy, [65]	Cohort,<br>100% post-menopausal women, 56.6% HTN, 32.1% current smokers.<br>Southern Italians	1,500, (unknown)	52.7 (5.7)	MDRD<br>*Reference*:<br>>60 (1,071)<br>*Comparison*:<br><60 (429)	Unspecified (65)	72.6	Incident<br>Fatal or non-fatal	Age, smoking, cholesterol, systolic blood pressure, fasting glucose, BMI, menopause, MS<br><br>(Continuous variable – SBP)
Protack 2011, USA, [66]	Cohort,<br>100% undergoing carotid revascularization, 32% DM, 87% HTN, 73% ever smokers.<br>Unknown ethnicity	921, (64)	71 (10)	MDRD<br>*Reference*:<br>>60 (604)<br>*Comparison*:<br>30-60 (262)<br><30 (55)	Unspecified (28)	1	Incident	Unadjusted
Pulli 2005, Italy, [67]	Cohort,<br>100% undergoing carotid revascularization	1,883, (2)	70 (7.3)	Serum Creatinine<br>*Reference*:<br>>30 (1,870)<br>*Comparison*:<br><30 (13)	Unspecified (48)	36	Incident	Age, sex, respiratory insufficiency, cardiac disease, re-intervention.<br><br>Not adjusted for hypertension.

Rahman 2006, USA, ALLHAT trial, [68]	RCT, *Inclusion criteria*: >55 years, HTN with ≥1 risk factor for coronary heart disease. *Intervention:* Chlorthalidone *Control:* Amlodipine/Lisinopril 36.1% DM, 21.9% smoking, 48% white/31% black	31,897, (54)	67.9 (7.8)	MDRD *Reference*: >90 (8,126) *Comparison*: 60-90 (18,109) 15-60 (5662)		Unspecified (1,435)	72	Incident or recurrent Fatal or non-fatal	(Continuous variable – SBP, DBP)
Ruilope 2001, Multinational, HOT study, [69]	RCT, *Inclusion criteria:* Hypertensive *Intervention:* Aspirin, DBP target ≤80/85/90mmHg *Control:* Placebo, DBP target ≤80/85/90mmHg, 8% DM, 15.9% smokers. Unknown ethnicity	18,790, (53)	61.5 (7.5)	Cockcroft Gault *Reference*: >60 (15,770) *Comparison*: <60 (2,821)		Unspecified (288)	45.6	Incident or recurrent Fatal or non-fatal	Blood pressure, age, sex, smoking, previous cardiovascular disease, diabetes, total serum cholesterol (Categorical variable – target DBP groups)
Ruilope 2007, Multinational, VALUE trial, [70]	RCT, *Inclusion criteria*: Hypertensive, high cardiovascular risk. *Intervention:* Valsartan *Control:* Amlodipine, 89% white/4% black/4% Asian. 31.7% DM, 24% smokers	15,245, (58)	67.2 (8.1)	Cockcroft Gault *Reference*: >60 (9,214); *Comparison*: <60 (5,999)	Dipstick *Reference*: None (11,788) *Comparison*: Any (3,435)	Unspecified (603)	45.6	Incident or recurrent Fatal or non-fatal	Age, sex, IHD, LVH, all-cause death. Not adjusted for hypertension.
Sandsmark 2015, USA, CRIC study, [71]	Cohort 55.2% DM, 33.9% CVD, 13.4% smoking. 42.4% White	3939 (55.2)	58.1 (10.9)	MDRD *Reference:* >60 (702) *Comparison:* 45-60 (1091) 30-44 (1339) <30 (807)	24h urine protein *Reference:* <0.1g/24h (1375) *Comparison:* 0.1-0.5 (1094) 0.5-1.5 (582)	Unspecified (143)	76.8	Incident	Age, sex, race, DM, SBP, hyperlipidaemia, smoking, alcohol use. (Continuous variable – baseline SBP, single reading)

					>1.5 (690)				
Shavit 2014,<br>Israel, [72]	Cohort<br>100% cardiac surgery,<br>81% HTN, 42% DM,<br>12% previous stroke	788 (60)	70 (10)	MDRD<br>*Reference:*<br>≥60 (none)<br>*Comparison:*<br><60 (788)		Unspecified (20)	0.5	Incident	Preoperative Hb, sex, severity of preop HF, DM, incidence of blood transfusion, type of surgery, use of diuretics, intraoperative inotrope use.<br><br>(Not adjusted for HTN)
Shih 2017,<br>Taiwan, [73]	Cohort<br>100% sepsis survivors,<br>87.5% HTN, 59.9% CAD, 58.7% DM	304902<br>(56.5)	67.4 (14.4)	MDRD<br>*Reference:*<br>≥60 (none)<br>*Comparison:*<br><60 (304902)		Ischemic (8352)	30	Incident	Subgroup analysis of HR: sex, age, Charlson comorbidity index, HTN, DM, CCF, CAD, stroke, number of organ failure, site of infection, ICU, shock, use of mechanical ventilator.<br><br>(Categorical variable)
Shimizu 2011,<br>Japan,<br>CIRCS study, [74]	Cohort,<br>3.7% DM, 14.1% HTN,<br>26.3% current smokers.<br>100% Asian	12,222,<br>(37)	53.5<br>(unknown)	Japan-specific<br>*Reference*:<br>>90 (4,131)<br>*Comparison*:<br>60-90 (6,340)<br><60 (1,309)		Unspecified (53)<br>Ischemic (327)<br>Hemorrhagic<br>(186)	204	Incident<br>Fatal or non-fatal	Family history of stroke, BMI, SBP, anti-hypertensives, smoking, ETOH, total cholesterol, diabetes, menopausal status.<br><br>(Continuous (SBP) and categorical (HTN) variables)

Shlipak 2001, USA, HERS study, [75]	RCT, *Inclusion criteria:* Post menopause, aged <80, known IHD, no hysterectomy. *Intervention:* HRT *Control:* Placebo 25% DM, 58.7% HTN, 13.4% current smokers. 89.2% White	2,763, (0)	66.5 (6.8)	Cockcroft Gault *Reference*: >60 (1,306) *Comparison*: 40-60 (1,135) <40 (322)		Unspecified (214)	49.2	Incident or recurrent Non-fatal	Age, race, hypertension, diabetes, smoking, previous CABG, BMI, waist: hip ratio, LDL/HDL cholesterol, TGs, lipoprotein(a) level, physical activity, lipid lowering medication and diuretic use, AF (categorical variable)
Sidawy 2008, USA, [76]	Cohort, 100% undergoing carotid artery intervention. 18% diabetics, 40% smokers, 9% previous CVA, 82% white	22,080 (98)	68.3 (8.6)	MDRD *Reference*: >60 (13,965) *Comparison*: 30-60 (6,423) <30 (511)		Unspecified (374)	1	Incident or recurrent Non-fatal	Age, sex, history of CVA, CCF, COPD, diabetes, smoking, ETOH, functional status, hemoglobin, albumin. Not adjusted for hypertension.
Synhaeve 2016, Netherland, FUTURE study, [77]	Cohort 100% stroke, 52.4% smoking, 35.2% HTN, 8.3% DM. Unknown ethnicity	460 (47.4)	41.2 (7.6)	CKD-Epi *Reference:* >120 (39) *Comparison:* 60-120 (392) <60 (29)		Unspecified (52)	138	Recurrent	Age, gender, HTN, DM, CVD. (Categorical variable – HTN = SBP≥135 or DBP≥85 or both – single reading)
Tonelli 2005, USA, CARE trial, [78]	RCT, *Inclusion criteria:* Hyperlipidemia and previous MI *Intervention:* Pravastatin *Control:* Placebo, 14.1% DM, 42.5% HTN, 16.1% current smokers. Unknown ethnicity	4,098, (86)	59.7 (50-70)	MDRD *Reference*: >60 (3,218) *Comparison*: <60 (880)	Dipstick *Reference*: None (3,546) *Comparison*: Macro (552)	Unspecified (130)	58.9	Incident Non-fatal	Age, sex, ethnic origin, smoking, BMI, waist: hip ratio, fasting glucose, hemoglobin, albumin, LDL/HDL cholesterol, TGs, systolic/diastolic blood pressure, location, LVEF, use of drugs (ACEi, aspirin, or pravastatin).

								(Continuous variables – SBP/DBP)
Usui 2017, Japan, Hisayama Study, [79]	Cohort<br>21.3% smoking, 12% DM	2630 (42.1)	58.9 (11.4)	CKD-Epi<br>*Reference:*<br>≥60 (2273)<br>*Comparison:*<br><60 (357)	Ischemic (212)<br>Hemorrhagic (65)	228	Incident	Age, sex, SBP, antihypertensive Rx, DM, BMI, serum albumin, serum hs-CRP, ECG abnormalities, smoking habit, alcohol intake, regular exercise.<br><br>(Cateogrical & continuous variables – on Rx, SBP)
Wang 2017, China, [80]	Cohort<br>100% stroke, 75.9% HTN, 43.1% smoking, 18.8% DM, 12.9% IHD	21075 (62.7)	64.3 (12)	CKD-Epi<br>*Reference:*<br>≥90 (11847)<br>*Comparison:*<br>60-89 (5292)<br><60 (1596)	Unspecified (916)	12	Recurrent	Age, sex, history of stroke, DM, dyslipidaemia, baseline NIHSS, current/previous smoking, alcohol, AF, CAD, BMI on admission & pneumonia. Stratified by HTN status.<br><br>(Categorical variable – self-reported history, on Rx before index event or new diagnosis at DC)

Weiner 2004, Multinational, ARIC, [81] Framingham, Framingham offspring and Cardiovascular health studies,	Cohort,<br>25% smokers, 9.5% DM, 39.7% HTN, 18% black	22,634, (44)	57.1 (11.6)	MDRD<br>*Reference*:<br>>60 (20,970)<br>*Comparison*:<br>15-60 (1,664)		Unspecified (712)	99	Incident or recurrent<br>Fatal or non-fatal	Age, sex, hypertension, diabetes, blood pressure, BMI, total /HDL cholesterol, smoking, ETOH, LVH, education, race<br><br>Hypertension was defined as systolic BP ≥140 mmHg, diastolic ≥90 mmHg, or use of an antihypertensive medication.<br><br>(Categorical [history of] and continuous [sbp] variables)
Yamamoto 2009, Japan, [82]	Cohort,<br>100% T2DM,<br>Unknown ethnicity	653, (65)		Japan-specific<br>*Reference*:<br>>60 (506)<br>*Comparison*:<br><60 (147)	ACR<br>*Reference*:<br>None (312)<br>*Comparison*:<br>Micro (341)	Unspecified (5)	36	Incident or recurrent<br>Fatal or non-fatal	Unadjusted
Yano 2011, Japan, [83]	Cohort,<br>100% hypertensive, 14.6% DM, 16.8% current smokers.<br>Unknown ethnicity	514, (37)	72.2 (8.6)	Cockcroft Gault<br>*Reference*:<br>>60 (289)<br>*Comparison*:<br><60 (225)		Unspecified (8) – ischemic (30) hemorrhagic (5)	41	Incident<br>Fatal or non-fatal	HTN = clinic BP ≥ 140/90 mmHg or on medication with anti-hypertensive drugs.<br><br>(Categorical variable – all patients were hypertensive but analysis was adjusted for antihypertensive med use)
Zhang 2015, China, CSPPT, [84]	RCT<br>*Inclusion criteria:*<br>45-75 yrs with HTN<br>*Intervention:*<br>Enalapril & folic acid<br>*Control:*<br>31% smoking, 11.1% DM	19599 (40.8)	60 (7.5)	CKD-Epi<br>*Reference:*<br>≥90 (13418)<br>*Comparison:*<br>60-89 (5768)<br><60 (413)	Urine dipstick<br>*Reference:*<br>None (16663)<br>*Comparison:*<br>Trace (1812)<br>≥1+ (1154)	Ischemic (472)<br>Hemorrhagic (111)<br>Undefined (2)	54	Incident	Age, study center, gender, treatment group, smoking, alcohol, BMI, baseline SBP/DBP, mean SBP/DBP over treatment period, TC, HDL, FPG,

									homocysteine, folate.<br><br>(Continuous variables – baseline & mean)
Zheng 2012, China, [85]	Cohort,<br>100% HTN, 11% diabetics, 39% smokers.<br>Mainly Chinese	3,711 (43)	56.3 (10.7)	CKD-EPI<br>*Reference*:<br>>90 (1625)<br>*Comparison*:<br>60-90 (1967)<br><60 (119)		Ischemic (98)<br>Hemorrhagic (75)	55.4	Incident<br>Fatal or non-fatal	Age, sex, ethnicity, BP, BMI, antihypertensive drug use, smoking, ETOH, DM, lipids, duration of HTN, lipid lower medications.<br><br>The mean of three BP measures was calculated and used for all analysis. Participants with hypertension at baseline were defined as they had an average systolic BP at least 140 mmHg, and/or an average diastolic BP at least 90 mmHg, and/or use of antihypertensive medications within the previous 2 weeks.<br><br>(Both continuous [SBP & DBP] and categorical variables [antihypertensive medication & duration > 10 years])

Abbreviations: AAA, abdominal aortic aneurysm; ACE, Angiotensin Converting Enzyme inhibitor; ACR, albumin:creatinine ratio; AF, atrial fibrillation; AR, aortic regurgitation; A2RB, Angiotensin 2 Receptor Blocker; BMI, body mass index; BMS, bare metal stent; Ca, calcium; CABG, coronary artery bypass grafting; CAD, coronary artery disease; CCF,

congestive cardiac failure; CEA, carotid endarterectomy; CLD, chronic liver disease; COPD, chronic obstructive pulmonary disease; CRP, C Reactive Protein; CVA, cerebrovascular accident; CVD, cardiovascular disease; DAPT, dual anti-platelet therapy; DBP, diastolic blood pressure; DM, diabetes mellitus; ECG, electrocardiograph; ETOH, alcohol; GFR, glomerular filtration rate; Hb, haemoglobin; HDL, high density lipoprotein; HIV, Human Immunodeficiency Virus; HRT, hormone replacement therapy; HTN, hypertension; IHD, ischemic heart disease; IS, ischemic stroke; LAD, left anterior descending artery; LDL, low density lipoprotein; LVEF, left ventricular ejection fraction; LVH, left ventricular hypertrophy; MDRD, Modification of Diet in Renal Disease; MI, myocardial infarction; NIHSS, National Institute of Health Stroke Scale; NSAIDs, Nonsteroidal anti-inflammatory drugs; NYHA, New York Heart Association; PCI, percutaneous coronary intervention; PCR, protein:creatinine ratio; PP, pulse pressure; PVD, peripheral vascular disease; Rx, treatment; SBP, systolic blood pressure; SD, standard deviation; SES, socio-economic status; STS score, Society of Thoracic Surgery score; TAVI, transcatheter aortic valve implantation; TC, total cholesterol; TE, thromboembolic; TG, triglyceride; TIA, transient ischemic attack

References to included studies:

1. Aguilar MI, O'Meara ES, Seliger S, Longstreth WT, Jr., Hart RG, Pergola PE, et al. Albuminuria and the risk of incident stroke and stroke types in older adults. *Neurology*. 2010;75:1343-1350
2. Banerjee A, Fauchier L, Vourc HP, Andres CR, Taillandier S, Halimi JM, et al. Renal impairment and ischemic stroke risk assessment in patients with atrial fibrillation: The Loire Valley Atrial Fibrillation project. *Journal of the American College of Cardiology*. 2013;61:2079-2087
3. Bansal N, McCulloch CE, Lin F, Robinson-Cohen C, Anderson AH, Xie D, et al. Different components of blood pressure are associated with increased risk of atherosclerotic cardiovascular disease versus heart failure in advanced chronic kidney disease. *Kidney International*. 2016;90:1348-1356
4. Bautista J, Bella A, Chaudhari A, Pekler G, Sapra KJ, Carbajal R, et al. Advanced chronic kidney disease in non-valvular atrial fibrillation: Extending the utility of R2CHADS2 to patients with advanced renal failure. *Clinical Kidney Journal*. 2015;8:226-231
5. Bax L, Algra A, Mali WPTM, Edlinger M, Beutler JJ, van der Graaf Y, et al. Renal function as a risk indicator for cardiovascular events in 3216 patients with manifest arterial disease. *Atherosclerosis*. 2008;200:184-190
6. Bedimo RJ, Westfall AO, Drechsler H, Vidiella G, Tebas P. Abacavir use and risk of acute myocardial infarction and cerebrovascular events in the highly active antiretroviral therapy era. *Clin. Infect. Dis.* 2011;53:84-91
7. Bos MJ, Koudstaal PJ, Hofman A, Breteler MMB. Decreased glomerular filtration rate is a risk factor for hemorrhagic but not for ischemic stroke: The Rotterdam Study. *Stroke*. 2007;38:3127-3132
8. Cea Soriano L, Johansson S, Stefansson B, Rodriguez LAG. Cardiovascular events and all-cause mortality in a cohort of 57,946 patients with type 2 diabetes: Associations with renal function and cardiovascular risk factors. *Cardiovascular Diabetology*. 2015;14:1-15
9. Cheng T-YD, Wen S-F, Astor BC, Tao XG, Samet JM, Wen CP. Mortality risks for all causes and cardiovascular diseases and reduced GFR in a middle-aged working population in taiwan. *Am. J. Kidney Dis.* 2008;52:1051-1060
10. Codner P, Levi A, Gargiulo G, Praz F, Hayashida K, Watanabe Y, et al. Impact of renal dysfunction on results of transcatheter aortic valve replacement outcomes in a large multicenter cohort. *American Journal of Cardiology*. 2016;118:1888-1896
11. Crimi G, Leonardi S, Costa F, Adamo M, Ariotti S, Valgimigli M. Role of stent type and of duration of dual antiplatelet therapy in patients with chronic kidney disease undergoing percutaneous coronary interventions. Is bare metal stent implantation still a justifiable choice? A post-hoc analysis of the all comer PRODIGY trial. *International Journal of Cardiology*. 2016;212:110-117
12. D'Ascenzo F, Moretti C, Salizzoni S, Bollati M, D'Amico M, Ballocca F, et al. 30 days and midterm outcomes of patients undergoing percutaneous replacement of aortic valve according to their renal function: A multicenter study. *International Journal of Cardiology*. 2013;167:1514-1518
13. De Leeuw PW, Thijs L, Birkenhager WH, Voyaki SM, Efstratopoulos AD, Fagard RH, et al. Prognostic significance of renal function in elderly patients with isolated systolic hypertension: Results from the Syst-Eur trial. *J. Am. Soc. Nephrol.* 2002;13:2213-2222
14. de Mattos AM, Prather J, Olyaei AJ, Shibagaki Y, Keith DS, Mori M, et al. Cardiovascular events following renal transplantation: Role of traditional and transplant-specific risk factors. *Kidney Int.* 2006;70:757-764
15. Devbhandari MP, Duncan AJ, Grayson AD, Fabri BM, Keenan DJM, Bridgewater B, et al. Effect of risk-adjusted, non-dialysis-dependent renal dysfunction on mortality and morbidity following coronary artery bypass surgery: A multi-centre study. *Eur. J. Cardiothorac. Surg.* 2006;29:964-970
16. Deo R, Fyr CLW, Fried LF, Newman AB, Harris TB, Angleman S, et al. Kidney dysfunction and fatal cardiovascular disease--an association independent of atherosclerotic events: Results from the health, aging, and body composition (Health ABC) study. *Am. Heart J.* 2008;155:62-68
17. Dong K, Huang X, Zhang Q, Yu Z, Ding J, Song H. A lower baseline glomerular filtration rate predicts high mortality and newly cerebrovascular accidents in acute ischemic stroke patients. *Medicine*. 2017;96: e5868
18. Dukkipati S, O'Neill WW, Harjai KJ, Sanders WP, Deo D, Boura JA, et al. Characteristics of cerebrovascular accidents after percutaneous coronary interventions. *J. Am. Coll. Cardiol.* 2004;43:1161-1167

19. Dumonteil N, Van Der Boon RMA, Tchetche D, Chieffo A, Van Mieghem NM, Marcheix B, et al. Impact of preoperative chronic kidney disease on short- and long-term outcomes after transcatheter aortic valve implantation: A Pooled-RotterdAm-Milano-Toulouse in Collaboration Plus (PRAGMATIC-plus) initiative substudy. *American Heart Journal*. 2013;165:752-760
20. Ferro CJ, Chue CD, De Belder MA, Moat N, Wendler O, Trivedi U, et al. Impact of renal function on survival after transcatheter aortic valve implantation (TAVI): An analysis of the UK TAVI registry. *Heart*. 2015;101:546-552
21. Ford I, Bezlyak V, Stott DJ, Sattar N, Packard CJ, Perry I, et al. Reduced glomerular filtration rate and its association with clinical outcome in older patients at risk of vascular events: Secondary analysis. *PLoS Med.* 2009;6:e1000016
22. Garcia-Carretero R, Vigil-Medina L, Barquero-Perez O, Goya-Esteban R, Mora-Jimenez I, Soguero-Ruiz C, et al. Cystatin C as a predictor of cardiovascular outcomes in a hypertensive population. *Journal of Human Hypertension*. 2017;31:801-807
23. Garcia-Gil M, Parramon D, Comas-Cufi M, Marti R, Ponjoan A, Alves-Cabratosa L, et al. Role of renal function in cardiovascular risk assessment: A retrospective cohort study in a population with low incidence of coronary heart disease. *Preventive Medicine*. 2016;89:200-206
24. Gelsomino S, Del Pace S, Parise O, Caciolli S, Matteucci F, Fradella G, et al. Impact of renal function impairment assessed by CKD-EPI estimated glomerular filtration rate on early and late outcomes after coronary artery bypass grafting. *International Journal of Cardiology*. 2017;227:778-787
25. Go AS, Fang MC, Udaltsova N, Chang Y, Pomernacki NK, Borowsky L, et al. Impact of proteinuria and glomerular filtration rate on risk of thromboembolism in atrial fibrillation: The anticoagulation and risk factors in atrial fibrillation (ATRIA) study. *Circulation*. 2009;119:1363-1369
26. Gruberg L, Jeremias A, Rundback JH, Anderson HV, Spertus JA, Kennedy KF, et al. Impact of glomerular filtration rate on clinical outcomes after carotid artery revascularization in 11,832 patients from the CARE registry. *Catheterization and Cardiovascular Interventions*. 2014;84:246-254
27. Guo Y, Wang H, Zhao X, Zhang Y, Zhang D, Ma J, et al. Sequential changes in renal function and the risk of stroke and death in patients with atrial fibrillation. *Int. J. Cardiol.* 2013;168:4678-4684
28. Holme I, Fayyad R, Faergeman O, Kastelein JJP, Olsson AG, Tikkanen MJ, et al. Cardiovascular outcomes and their relationships to lipoprotein components in patients with and without chronic kidney disease: Results from the IDEAL trial. *J. Intern. Med.* 2010;267:567-575
29. Holzmann MJ, Aastveit A, Hammar N, Jungner I, Walldius G, Holme I. Renal dysfunction increases the risk of ischemic and hemorrhagic stroke in the general population. *Ann. Med.* 2012;44:607-615
30. Hwang HS, Park MW, Yoon HE, Chang YK, Yang CW, Kim SY, et al. Clinical significance of chronic kidney disease and atrial fibrillation on morbidity and mortality in patients with acute myocardial infarction. *American Journal of Nephrology*. 2014;40:345-352
31. Irie F, Iso H, Sairenchi T, Fukasawa N, Yamagishi K, Ikehara S, et al. The relationships of proteinuria, serum creatinine, glomerular filtration rate with cardiovascular disease mortality in japanese general population. *Kidney Int.* 2006;69:1264-1271
32. Itaya H, Shiba M, Joki N, Nakamura M. Combined assessment of chronic kidney disease and subclinical peripheral artery disease used to predict future cardiac events. *Nephrology*. 2010;15:230-235
33. Ix JH, Mercado N, Shlipak MG, Lemos PA, Boersma E, Lindeboom W, et al. Association of chronic kidney disease with clinical outcomes after coronary revascularization: The arterial revascularization therapies study (ARTS). *Am. Heart J.* 2005;149:512-519
34. John R, Choudhri AF, Weinberg AD, Ting W, Rose EA, Smith CR, et al. Multicenter review of preoperative risk factors for stroke after coronary artery bypass grafting. *Ann. Thorac. Surg.* 2000;69:30-35
35. Kokubo Y, Nakamura S, Okamura T, Yoshimasa Y, Makino H, Watanabe M, et al. Relationship between blood pressure category and incidence of stroke and myocardial infarction in an urban japanese population with and without chronic kidney disease: The Suita study. *Stroke*. 2009;40:2674-2679
36. Konishi H, Kasai T, Miyauchi K, Kajimoto K, Kubota N, Dohi T, et al. Association of low glomerular filtration rate with the incidence of stroke in patients following complete coronary revascularization. *Circ. J.* 2011;75:2372-2378

37. Kooiman J, Van Rein N, Spaans B, Van Beers KAJ, Bank JR, Van De Peppel WR, et al. Efficacy and safety of vitamin k-antagonists (VKA) for atrial fibrillation in non-dialysis dependent chronic kidney disease. *PLoS ONE*. 2014;9:e94420
38. Koren-Morag N, Goldbourt U, Tanne D. Renal dysfunction and risk of ischemic stroke or tia in patients with cardiovascular disease. *Neurology*. 2006;67:224-228
39. Kovesdy CP, Alrifai A, Gosmanova EO, Lu JL, Canada RB, Wall BM, et al. Age and outcomes associated with BP in patients with incident CKD. *Clinical Journal of the American Society of Nephrology*. 2016;11:821-831
40. Kurth T, de Jong PE, Cook NR, Buring JE, Ridker PM. Kidney function and risk of cardiovascular disease and mortality in women: A prospective cohort study. *BMJ*. 2009;338:b2392
41. Kushiro T, Kario K, Saito I, Teramukai S, Sato Y, Okuda Y, et al. Increased cardiovascular risk of treated white coat and masked hypertension in patients with diabetes and chronic kidney disease: The HONEST study. *Hypertension Research*. 2017;40:87-95
42. Kuwashiro T, Sugimori H, Ago T, Kamouchi M, Kitazono T. Risk factors predisposing to stroke recurrence within one year of non-cardioembolic stroke onset: The Fukuoka Stroke Registry. *Cerebrovasc. Dis.* 2012;33:141-149
43. Lee M, Markovic D, Ovbiagele B. Impact and interaction of low estimated gfr and b vitamin therapy on prognosis among ischemic stroke patients: The vitamin intervention for stroke prevention (VISP) trial. *Am. J. Kidney Dis.* 2013;62:52-57
44. Lee SJ, Lee DG. Relationship between kidney dysfunction and ischemic stroke outcomes: Albuminuria, but not estimated glomerular filtration rate, is associated with the risk of further vascular events and mortality after stroke. *PLoS ONE*. 2016;11:e0155939.
45. Li Z, Wang A, Cai J, Gao X, Zhou Y, Luo Y, et al. Impact of proteinuria and glomerular filtration rate on risk of ischaemic and intracerebral hemorrhagic stroke: A result from the Kailuan study. *European Journal of Neurology*. 2015;22:355-360
46. Luo Y, Wang X, Wang Y, Wang C, Wang H, Wang D, et al. Association of glomerular filtration rate with outcomes of acute stroke in type 2 diabetic patients: Results from the China National Stroke Registry. *Diabetes Care*. 2014;37:173-179
47. Mann JF, Gerstein HC, Pogue J, Bosch J, Yusuf S. Renal insufficiency as a predictor of cardiovascular outcomes and the impact of ramipril: The HOPE randomized trial. *Ann. Intern. Med.* 2001;134:629-636
48. Mann JFE, Sheridan P, McQueen MJ, Held C, Arnold JMO, Fodor G, et al. Homocysteine lowering with folic acid and b vitamins in people with chronic kidney disease--results of the renal Hope-2 study. *Nephrology Dialysis Transplantation*. 2008;23:645-653
49. Marui A, Okabayashi H, Komiya T, Tanaka S, Furukawa Y, Kita T, et al. Impact of occult renal impairment on early and late outcomes following coronary artery bypass grafting. *Interactive Cardiovascular and Thoracic Surgery*. 2013;17:638-643
50. Matsue Y, Matsumura A, Abe M, Ono M, Seya M, Nakamura T, et al. Prognostic implications of chronic kidney disease and anemia after percutaneous coronary intervention in acute myocardial infarction patients. *Heart and Vessels*. 2013;28:19-26
51. McAlister FA, Wiebe N, Jun M, Sandhu R, James MT, McMurtry MS, et al. Are existing risk scores for nonvalvular atrial fibrillation useful for prediction or risk adjustment in patients with chronic kidney disease? *Canadian Journal of Cardiology*. 2017;33:243-252
52. McMullan CJ, Yano Y, Bakris GL, Kario K, Phillips RA, Forman JP. Racial impact of diurnal variations in blood pressure on cardiovascular events in chronic kidney disease. *Journal of the American Society of Hypertension*. 2015;9:299-306
53. Miceli A, Bruno VD, Capoun R, Romeo F, Angelini GD, Caputo M. Occult renal dysfunction: A mortality and morbidity risk factor in coronary artery bypass grafting surgery. *J. Thorac. Cardiovasc. Surg.* 2011;141:771-776
54. Muntner P, Judd SE, McClellan W, Meschia JF, Warnock DG, Howard VJ. Incidence of stroke symptoms among adults with chronic kidney disease: Results from the reasons for geographic and racial differences in stroke (REGARDS) study. *Nephrology Dialysis Transplantation*. 2012;27:166-173
55. Nagai K, Yamagata K, Ohkubo R, Saito C, Asahi K, Iseki K, et al. Annual decline in estimated glomerular filtration rate is a risk factor for cardiovascular events independent of proteinuria. *Nephrology*. 2014;19:574-580
56. Nakagawa K, Hirai T, Takashima S, Fukuda N, Ohara K, Sasahara E, et al. Chronic kidney disease and CHADS(2) score independently predict cardiovascular events and mortality in patients with nonvalvular atrial fibrillation. *Am. J. Cardiol.* 2011;107:912-916

57. Nakamura H, Mizuno K, Ohashi Y, Yoshida T, Hirao K, Uchida Y, et al. Pravastatin and cardiovascular risk in moderate chronic kidney disease. *Atherosclerosis*. 2009;206:512-517
58. Nakayama M, Metoki H, Terawaki H, Ohkubo T, Kikuya M, Sato T, et al. Kidney dysfunction as a risk factor for first symptomatic stroke events in a general japanese population--the Ohasama study. *Nephrology Dialysis Transplantation*. 2007;22:1910-1915
59. Nickolas TL, Khatri M, Boden-Albala B, Kiryluk K, Luo X, Gervasi-Franklin P, et al. The association between kidney disease and cardiovascular risk in a multiethnic cohort: Findings from the northern manhattan study (NOMAS). *Stroke*. 2008;39:2876-2879
60. Ninomiya T, Kiyohara Y, Kubo M, Tanizaki Y, Doi Y, Okubo K, et al. Chronic kidney disease and cardiovascular disease in a general japanese population: The Hisayama study. *Kidney Int.* 2005;68:228-236
61. Ohsawa M, Tanno K, Itai K, Turin TC, Okamura T, Ogawa A, et al. Comparison of predictability of future cardiovascular events between chronic kidney disease (CKD) stage based on ckd epidemiology collaboration equation and that based on modification of diet in renal disease equation in the Japanese general population - Iwate KENCO study. *Circulation Journal*. 2013;77:1315-1325
62. Papademetriou V, Zaheer M, Doumas M, Lovato L, Applegate WB, Tsioufis C, et al. Cardiovascular outcomes in action to control cardiovascular risk in diabetes: Impact of blood pressure level and presence of kidney disease. *American Journal of Nephrology*. 2016;43:271-280
63. Papademetriou V, Nylen ES, Doumas M, Probstfield J, Mann JFE, Gilbert RE, et al. Chronic kidney disease, basal insulin glargine, and health outcomes in people with dysglycemia: The origin study. *American Journal of Medicine*. 2017;130:1465.e27-1465.e39
64. Patel AD, Ibrahim M, Swaminathan RV, Minhas IU, Kim LK, Venkatesh P, et al. Five-year mortality outcomes in patients with chronic kidney disease undergoing percutaneous coronary intervention. *Catheterization and Cardiovascular Interventions*. 2017;89:E124-E132
65. Perticone F, Sciacqua A, Maio R, Perticone M, Laino I, Bruni R, et al. Renal function predicts cardiovascular outcomes in southern Italian postmenopausal women. *Eur. J. Cardiovasc. Prev. Rehabil.* 2009;16:481-486
66. Protack CD, Bakken AM, Saad WE, Davies MG. Influence of chronic renal insufficiency on outcomes following carotid revascularization. *Arch. Surg.* 2011;146:1135-1141
67. Pulli R, Dorigo W, Barbanti E, Azas L, Pratesi G, Innocenti AA, et al. Does the high-risk patient for carotid endarterectomy really exist? *Am. J. Surg.* 2005;189:714-719
68. Rahman M, Pressel S, Davis BR, Nwachuku C, Wright JT, Jr., Whelton PK, et al. Cardiovascular outcomes in high-risk hypertensive patients stratified by baseline glomerular filtration rate. *Ann. Intern. Med.* 2006;144:172-180
69. Ruilope LM, Salvetti A, Jamerson K, Hansson L, Warnold I, Wedel H, et al. Renal function and intensive lowering of blood pressure in hypertensive participants of the hypertension optimal treatment (HOT) study. *J. Am. Soc. Nephrol.* 2001;12:218-225
70. Ruilope LM, Zanchetti A, Julius S, McInnes GT, Segura J, Stolt P, et al. Prediction of cardiovascular outcome by estimated glomerular filtration rate and estimated creatinine clearance in the high-risk hypertension population of the VALUE trial. *J. Hypertens.* 2007;25:1473-1479
71. Sandsmark DK, Messe SR, Zhang X, Roy J, Nessel L, Lee Hamm L, et al. Proteinuria, but Not eGFR, Predicts Stroke Risk in Chronic Kidney Disease: Chronic Renal Insufficiency Cohort Study. *Stroke.* 2015;46:2075-2080
72. Shavit L, Hitti S, Silberman S, Tauber R, Merin O, Lifschitz M, et al. Preoperative hemoglobin and outcomes in patients with CKD undergoing cardiac surgery. *Clinical Journal of the American Society of Nephrology*. 2014;9:1536-1544
73. Shih CJ, Chao PW, Ou SM, Chen YT. Long-term risk of cardiovascular events in patients with chronic kidney disease who have survived sepsis: A nationwide cohort study. *Journal of the American Heart Association*. 2017;6: e004613
74. Shimizu Y, Maeda K, Imano H, Ohira T, Kitamura A, Kiyama M, et al. Chronic kidney disease and drinking status in relation to risks of stroke and its subtypes: The Circulatory Risk in Communities Study (CIRCS). *Stroke*. 2011;42:2531-2537
75. Shlipak MG, Simon JA, Grady D, Lin F, Wenger NK, Furberg CD, et al. Renal insufficiency and cardiovascular events in postmenopausal women with coronary heart disease. *J. Am. Coll. Cardiol.* 2001;38:705-711
76. Sidawy AN, Aidinian G, Johnson III ON, White PW, DeZee KJ, Henderson WG. Effect of chronic renal insufficiency on outcomes of carotid endarterectomy. *J. Vasc. Surg.* 2008;48:1423-1430

77. Synhaeve NE, Van Alebeek ME, Arntz RM, Maaijwee NAM, Rutten-Jacobs LCA, Schoonderwaldt HC, et al. Kidney dysfunction increases mortality and incident events after young stroke: The FUTURE study. *Cerebrovascular Diseases*. 2016;42:224-231
78. Tonelli M, Jose P, Curhan G, Sacks F, Braunwald E, Pfeffer M, et al. Proteinuria, impaired kidney function, and adverse outcomes in people with coronary disease: Analysis of a previously conducted randomised trial. *BMJ*. 2006;332:1426
79. Usui T, Nagata M, Hata J, Mukai N, Hirakawa Y, Yoshida D, et al. Serum non-high-density lipoprotein cholesterol and risk of cardiovascular disease in community dwellers with chronic kidney disease: The Hisayama study. *Journal of Atherosclerosis and Thrombosis*. 2017;24:706-715
80. Wang X, Wang Y, Patel UD, Barnhart HX, Li Z, Li H, et al. Comparison of associations of reduced estimated glomerular filtration rate with stroke outcomes between hypertension and no hypertension. *Stroke*. 2017;48:1691-1694
81. Weiner DE, Tighiouart H, Amin MG, Stark PC, MacLeod B, Griffith JL, et al. Chronic kidney disease as a risk factor for cardiovascular disease and all-cause mortality: A pooled analysis of community-based studies. *J. Am. Soc. Nephrol.* 2004;15:1307-1315
82. Yamamoto R, Kanazawa A, Shimizu T, Hirose T, Tanaka Y, Kawamori R, et al. Association between atherosclerosis and newly classified chronic kidney disease stage for Japanese patients with type 2 diabetes. *Diabetes Res. Clin. Pract.* 2009;84:39-45
83. Yano Y, Hoshide S, Etoh T, Tamaki N, Yokota N, Kario K. Synergistic effect of chronic kidney disease and high circulatory norepinephrine level on stroke risk in Japanese hypertensive patients. *Atherosclerosis*. 2011;219:273-279
84. Zhang C, Wang X, He M, Qin X, Tang G, Wang Y, et al. Proteinuria is an independent risk factor for first incident stroke in adults under treatment for hypertension in China. *Journal of the American Heart Association*. 2015;4:e002639
85. Zheng L, Sun Z, Zhang X, Li J, Hu D, Sun Y. The association between glomerular filtration rate and stroke in hypertensive patients in rural areas of China. *J. Hypertens.* 2012;30:901-907

Appendix Table III. Characteristics of studies included in the meta-analysis

Characteristics	Number of studies, total = 85<br>N	%
**Study**		
**Design**		
Randomized controlled trial	18	21.2
Cohort study	67	78.8
**Location**		
North America	24	28.2
Europe	16	18.8
Asia	31	36.5
Multinational	14	16.5
**Number of participants**		
0 to <2500	29	34.1
≥2500 to <5000	18	21.2
≥5000 to <20000	19	22.4
≥20000	19	22.4
**Duration of follow-up (months)**		
0 to <24	18	21.2
≥24 to <60	35	41.2
≥60 to <96	14	16.5
≥96	16	18.8
**Decade of publication**		
1970s	1	1.2
1980s	0	0
1990s	0	0
2000s	31	36.5
2010s	53	62.4
**Participant**		
**Mean age (years)**		
<60	22	25.9
≥60 to <65	17	20
≥65 to <70	25	29.4
≥70	20	23.5
**Hypertensives (%)**		
<25	5	5.9
≥25 to <50	19	22.4
≥50 to <75	27	31.8
≥75	25	29.4
**Diabetics (%)**		
<15	23	27.1
≥15 to <30	29	34.1
≥30	27	31.8
**Time of high stroke risk**		
Undergoing cardiac procedure	17	20
Undergoing carotid intervention	4	4.7
**Stroke**		
**Subtype**		
Unspecified	63	74.1
Ischemic	29	34.1
Hemorrhagic	17	20

Appendix Figure I: Unadjusted risk ratio (RR) for the association of CKD (defined as eGFR < 60 ml/min/1.73m^2) and stroke risk

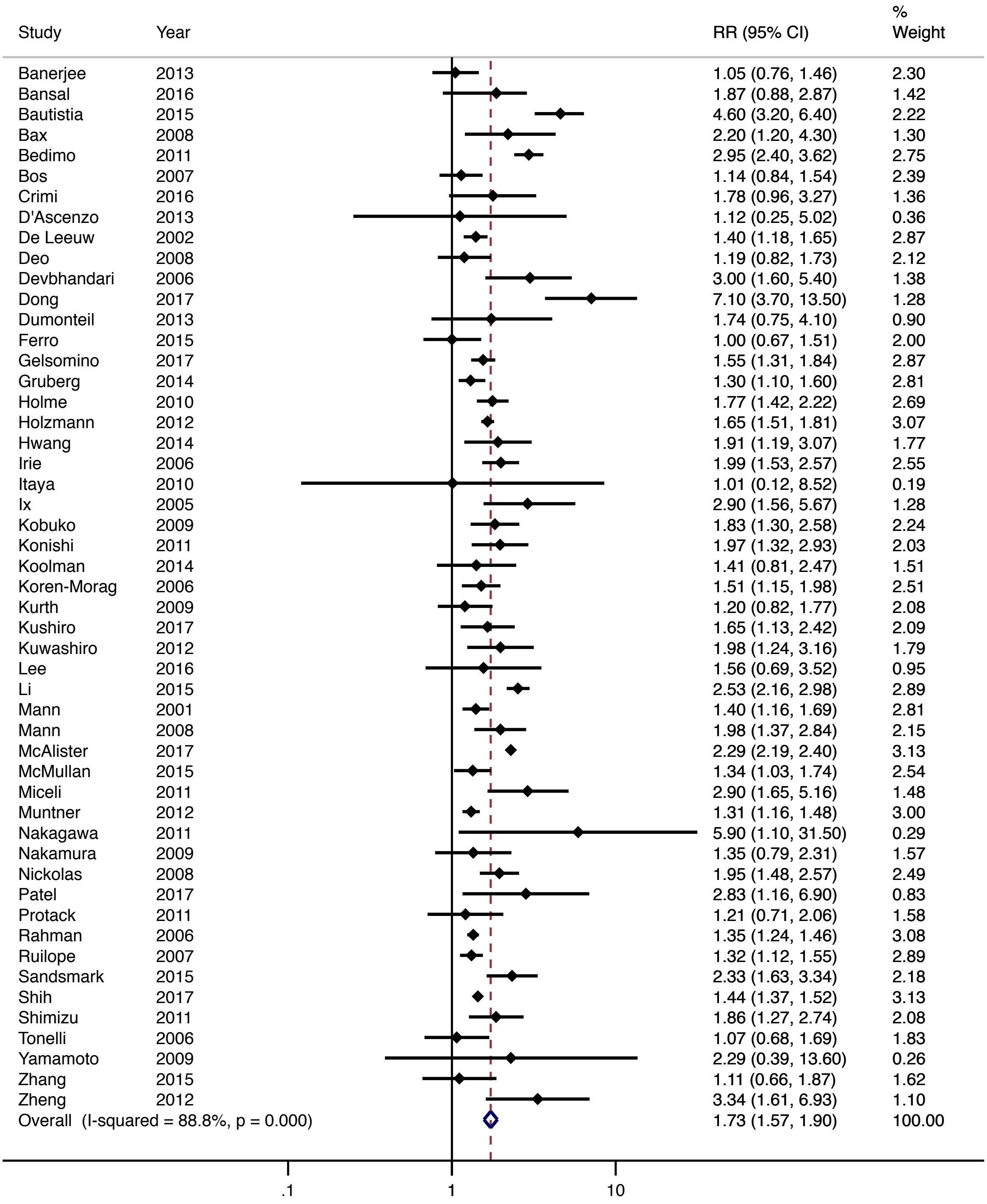

Appendix Figure II: Risk ratio (RR) for the association of CKD (defined as eGFR < 60 ml/min/1.73m^2) and stroke risk adjusted for traditional cardiovascular risk factors

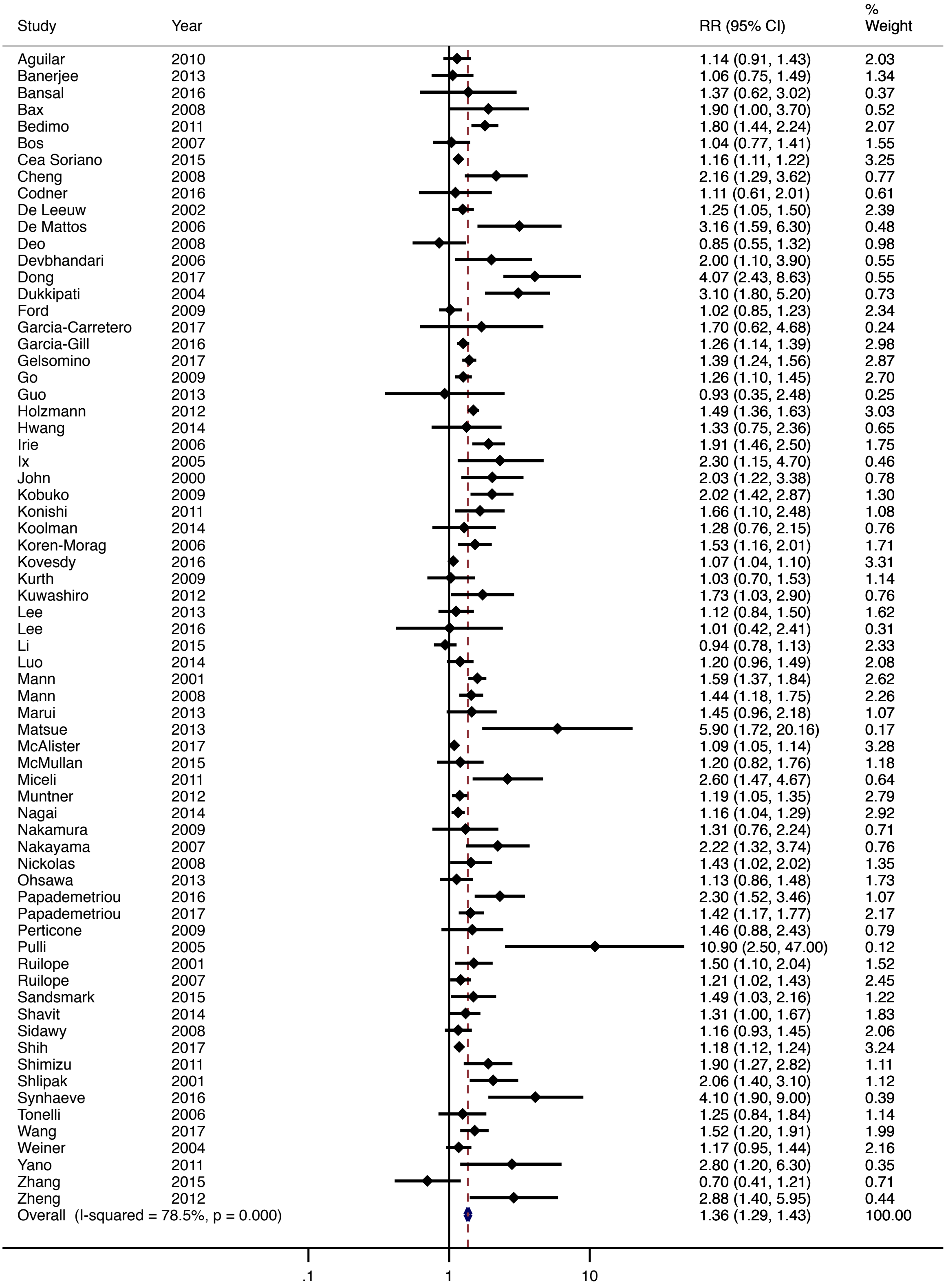

Appendix Figure III: Risk ratio (RR) for the association of CKD (defined as eGFR < 60 ml/min/1.73m^2) and stroke risk adjusted for traditional cardiovascular risk factors, using a fixed-effects model

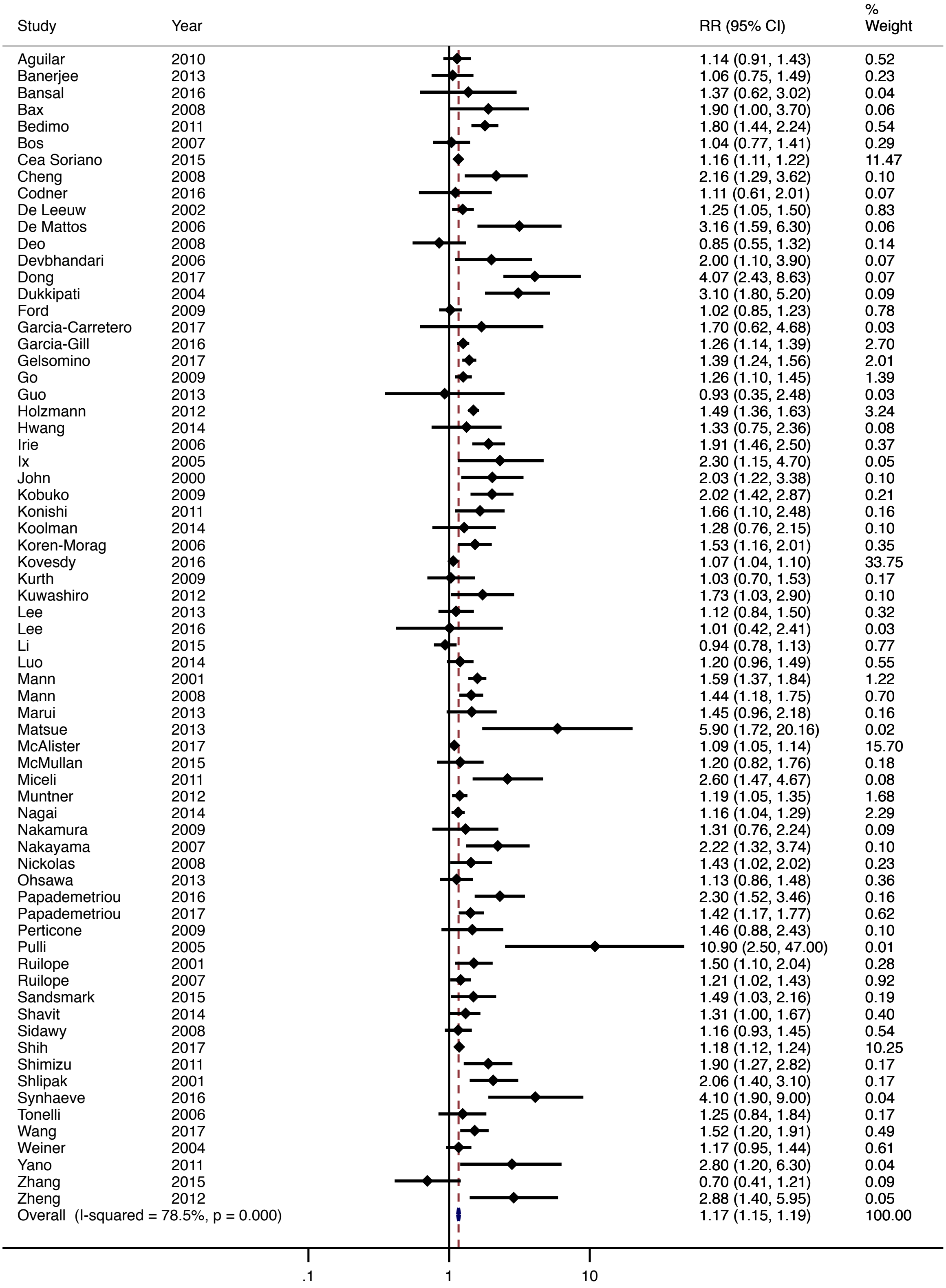

Appendix Figure IV: Risk ratio (RR) for the association of CKD and ischemic stroke risk adjusted for traditional cardiovascular risk factors

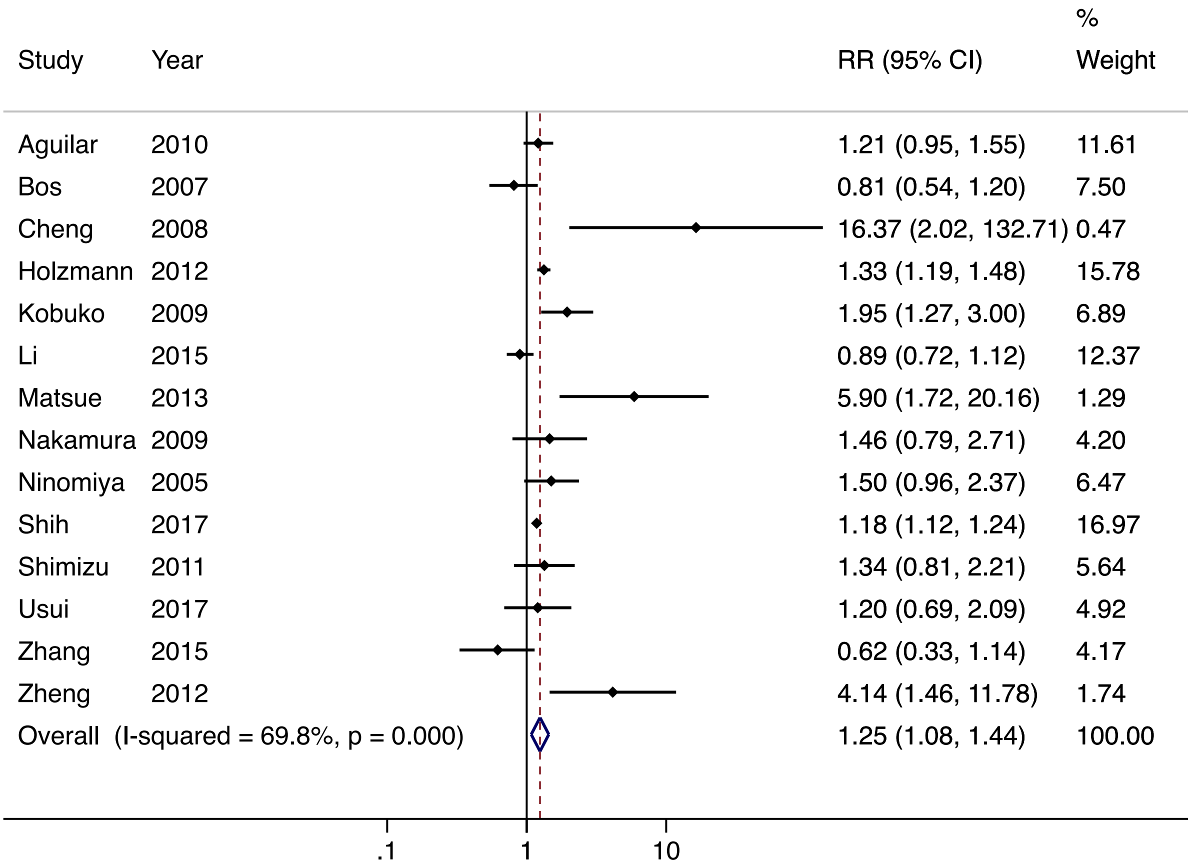

Appendix Figure V: Risk ratio (RR) for the association of CKD and hemorrhagic stroke risk adjusted for traditional cardiovascular risk factors

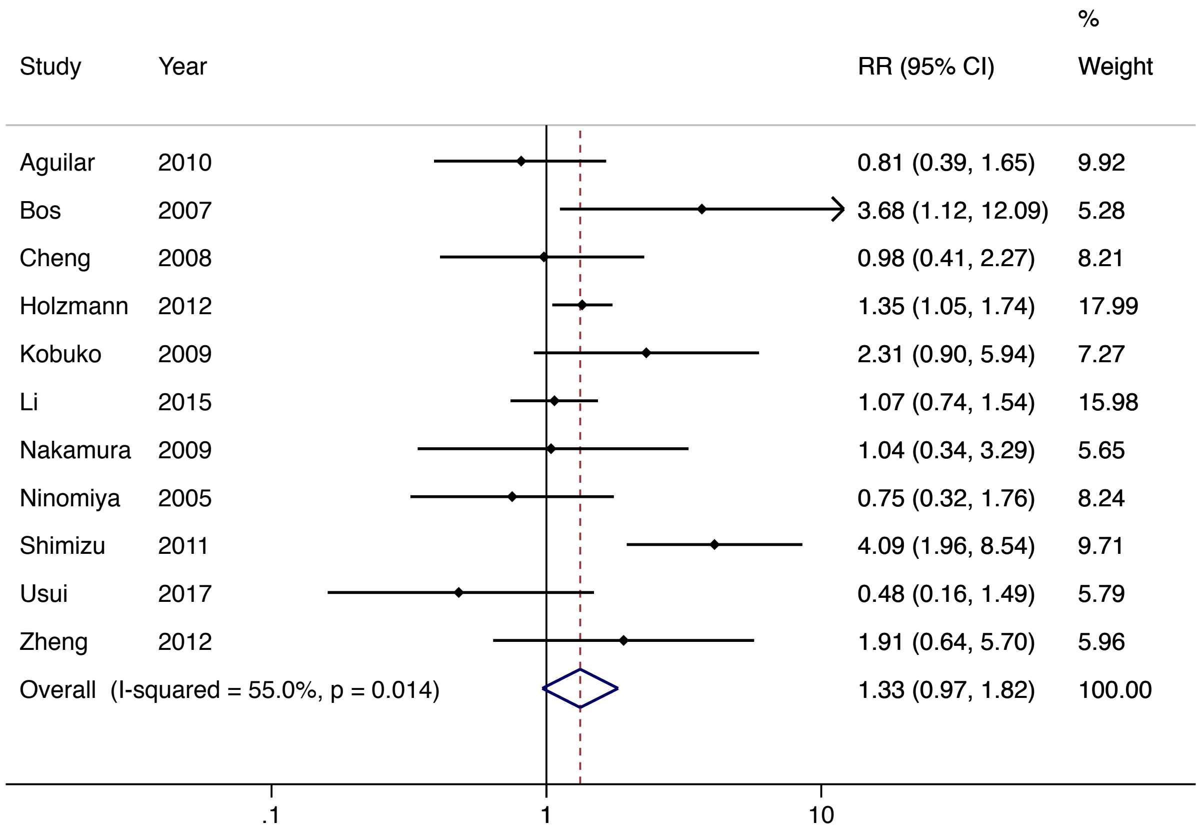

Appendix Figure VI: Funnel plot to assess for publication bias

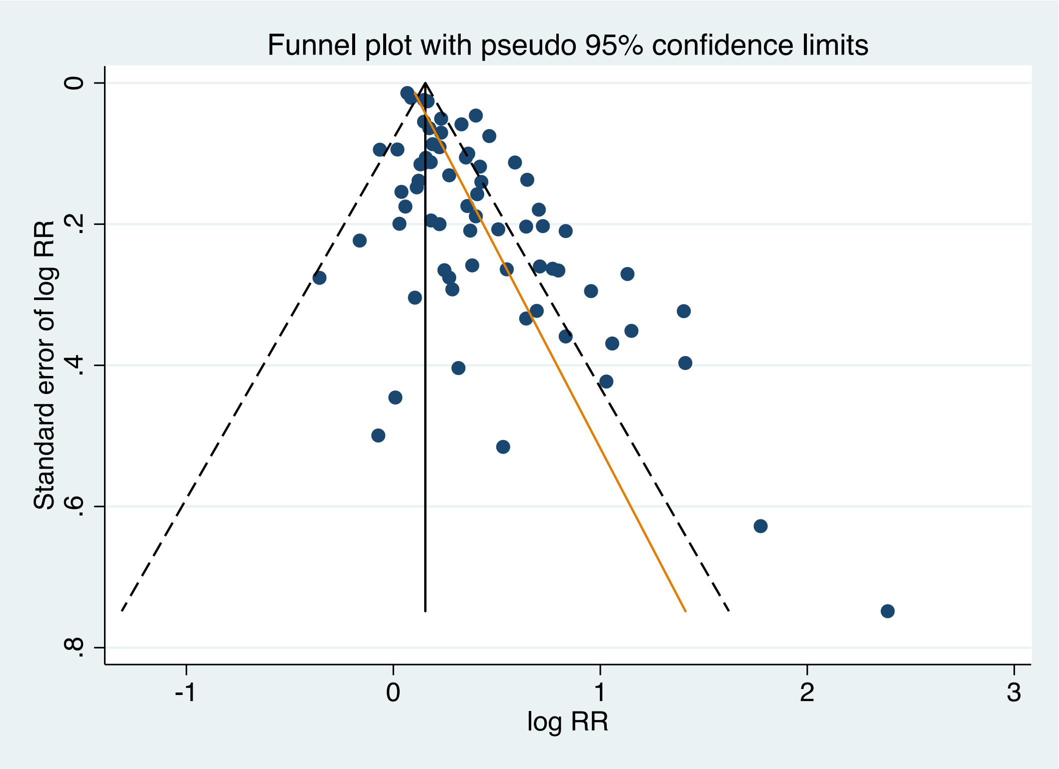

Appendix Table IV. Studies categorized according to a hierarchy of hypertension adjustment, from least (1) to best (4) adjustment

**1 = Baseline blood pressure at study entry**	**2= History of hypertension and/or on treatment and/or baseline blood pressure at study entry**	**3= History of hypertension and/or on treatment**	**4 = Multiple blood pressure readings over time**
De Mattos 2006[14]<br>Devbhandari 2006[15]<br>Garcia-Gill 2016[23]<br>Irie 2006[31]<br>Kobuko 2009[35]<br>Ohsawa 2013[61]<br>Perticone 2009[65]<br>Sandsmark 2015[71]<br>Synhaeve 2016[77]<br>Tonelli 2005[78]	Bansal 2016[3]<br>Bax 2008[5]<br>Bos 2007[7]<br>Cheng 2008[9]<br>Deo 2008[16]<br>Ford 2009[21]<br>Guo 2013[27]<br>Ix 2005[33]<br>Konishi 2011[36]<br>Koren-Morag 2006[38]<br>Kurth 2009[40]<br>Kuwashiro 2012[42]<br>Lee 2016[44]<br>Li 2015[45]<br>Luo 2014[46]<br>Mann 2001[47]<br>Mann 2008[48]<br>Matsue 2013[50]<br>Muntner 2012[54]<br>Nagai 2014[55]<br>Nakayama 2007[58]<br>Papademetriou 2016[62]<br>Papademetriou 2017[63]<br>Shimizu 2011[74]<br>Weiner 2004[81]<br>Zheng 2012[85]	Banerjee 2013[2]<br>Bedimo 2011[6]<br>Cea Soriano 2015[8]<br>Dukkipati 2004[18]<br>Go 2009[25]<br>Hwang 2014[30]<br>John 2000[34]<br>Kooiman 2014[37]<br>Marui 2013[49]<br>McAlister 2017[51]<br>Micelli 2011[53]<br>Nakamura 2009[57]<br>Nickolas 2008[59]<br>Shih 2017[73]<br>Shlipak 2001[75]<br>Wang 2017[80]<br>Yano 2011[83]	Anguilar 2010[1]<br>De Leeuw 2002[13]<br>Kovesedy 2016[39]<br>Lee 2013[43]<br>McMullan 2014[52]<br>Zhang 2015[84]
